# The spectrum of immunoglobulin heavy chain enhancer hijacking in chronic lymphocytic leukemia

**DOI:** 10.1038/s41375-026-02902-9

**Published:** 2026-04-23

**Authors:** Cosima Drewes, Cristina López, Nnamdi Okeke, Billy Jebaraj, Christoph Wiegreffe, Isabelle Kraus, Sina Hillebrecht, Amani Awada, Susanne Bens, Emil Chteinberg, Barbara Eichhorst, Sarah Datismann, Martin J. S. Dyer, Anja Fischer, Kirsten Fischer, Selina Glaser, Michael Hallek, Helene Kretzmer, Anja Mottok, Dominick Pfaff, Karoline Schnitzler, Jan P. Meier-Kolthoff, Matthias Schlesner, Christof Schneider, Stefan Britsch, Ole Ammerpohl, Stephan Stilgenbauer, Eugen Tausch, Reiner Siebert

**Affiliations:** 1 Institute of Human Genetics, Ulm University and Ulm University Medical Center, Ulm, Germany; 2 Institut d’Investigaciones Biomèdiques August Pi i Sunyer (IDIBAPS), Barcelona, Spain; 3 Hematopathology Section, Pathology Department, Hospital Clínic de Barcelona, Barcelona, Spain; 4 Departament de Fonaments Clínics, Universitat de Barcelona, Barcelona, Spain; 5 Division of CLL, Department of Internal Medicine III, Ulm University Medical Center, Ulm, Germany; 6 Institute of Molecular and Cellular Anatomy, Albert-Einstein-Allee 11, 89081 Ulm, Germany; 7 Max Planck Institute for Molecular Genetics, Berlin, Germany; 8 Digital Health Cluster, Hasso Plattner Institute for Digital Engineering, Digital Engineering Faculty, University of Potsdam, Potsdam, Germany; 9 Department I of Internal Medicine and German CLL Study Group; Center for Integrated Oncology Aachen Bonn Cologne Duesseldorf (CIO ABCD), University of Cologne, Faculty of Medicine and University Hospital of Cologne, Cologne, Germany; 10 The Ernest and Helen Scott Haematological Research Institute, Leicester Cancer Research Centre, University of Leicester, Leicester, UK; 11 Biomedical Informatics, Data Mining and Data Analytics, Faculty of Applied Computer Science and Medical Faculty, University of Augsburg, Augsburg, Germany; 12 Augsburg Bioinformatics Core Facility, University of Augsburg, Augsburg, Germany; 13 German Center for Child and Adolescent Health (DZKJ), partner site Ulm, Ulm, Germany

**Keywords:** Chronic lymphocytic leukaemia, Cancer genomics

## Abstract

Activation of oncogenes by hijacking immunoglobulin gene loci (*IG*) enhancers via chromosomal translocation is a common pathogenetic mechanism in B-cell malignancies, affecting 5–10% of chronic lymphocytic leukemia (CLL). The oncogenic partners in many of these cases remain unidentified. Therefore, we conducted a comprehensive analysis of 144 CLL samples with *IGH-*translocation excluding *IGH*::*BCL2*, *IGH*::*CCND1*, *IGH*::*BCL3* and *IGH*::*MYC*. By combining fluorescence in situ hybridization (FISH) with whole-genome, targeted sequencing, and RNA expression profiling, we identified 25 *IG*-translocation partners; 12 were previously unreported. Of 142 cases, 107 (75%) displayed an unmutated *IGHV*. Genetic profiling showed a heterogenous distribution of chromosomal aberrations and recurrently mutated genes across the groups. Of 41 informative cases, 32 (78%) exhibited breakpoints driven by aberrant class-switch recombination (CSR), with prominent involvement of *IGHM* (9/41) and *IGHG3* (9/41). Three cases with unmutated *IGHV* carried a juxtaposition of the *IGH* locus 5’ to the intact *NKX2.6* gene in chromosome 8p21.2 due to illegitimate VDJ recombination, associated with significant ectopic upregulation of *NKX2.6* transcriptional expression (FDR < 0.001, logFC: 15). Similarly, *METRNL*, located at the telomere of chromosome 17q25, was identified as a translocation partner gene in four cases. Our findings expand the spectrum of the oncogenic translocation partners targeting *IGH* in CLL.

## Introduction

In B-cell malignancies, chromosomal translocations involving immunoglobulin *(IG)* loci, particularly those targeting the *IGH* locus at 14q32, are a common mechanism of oncogene deregulation. The juxtaposition of the *IGH* locus with an oncogene can hijack *IG* enhancer elements and drive aberrant partner gene expression promoting malignancy [1]. In various precursor and mature B-cell malignancies, the identification of such oncogenes through characterization of *IG*-translocations has contributed not only to the biological and clinical subtyping of the neoplasms but also to the characterization of key pathways involved in tumorigenesis [2–7]. While chromosomal translocations involving band 14q32 suggesting a breakpoint affecting the *IGH* locus can be recurrently detected by conventional cytogenetic analysis in several mature B-cell malignancies such as mantle cell lymphoma, follicular lymphoma or Burkitt lymphoma, the cytogenetic analysis of CLL cells has been historically more challenging due to the lower proliferative capacity of the tumor cells in vitro resulting in no or few tumor metaphases particularly in unstimulated cultures [8, 9]. Therefore, chromosomal translocations have been previously detected with reduced sensitivity in CLL. This issue has been resolved more recently by supplementing the culture with the immunostimulatory CpG-oligonucleotide DSP30 and interleukin 2, which enhanced metaphase generation [10–12]. Nevertheless, to adjust for the comparably lower yield of metaphases in CLL, interphase cytogenetics by FISH has been increasingly used to identify *IGH*-translocations in CLL [9, 13]. However, when interphase FISH is employed, the identification of the translocation partner is usually limited by the knowledge of potential partners.

Using FISH, *IG*-translocations occur in approximately 8% of CLL cases, predominantly involving the *IGH* locus, and less frequently (each <1%) the light chain *IGK* and *IGL* loci [1]. *BCL2* and *BCL3* are among the most common translocation partners in CLL with 2% and less than 1% of all CLL cases, respectively [1, 14, 15]. Generally, more than 70% of the CLL cases with *IGH*-translocations carry an unmutated *IGHV*. This strong though not perfect association between *IGH*-translocation and mutation might also account in part for the more unfavorable prognosis related to the presence of *IGH*-translocations in CLL [16].

Despite recent advances in the characterization of the mutational landscape of CLL, the spectrum of oncogenes deregulated by *IGH* enhancer hijacking has not yet been well defined and many *IGH*-translocation partners remain unidentified [1]. The present study aims at characterizing the *IGH*-partners and mutational landscape of CLL samples with *IGH*-translocation other than *BCL2*, *CCND1*, *BCL3* and *MYC* in a cohort of 144 samples defined by FISH.

## Methods

### Patient and control samples

144 patient samples with FISH-proven *IGH*-break and unknown partner from the biobank of the Department of Internal Medicine 3, University Medical Center Ulm were studied. Cases with proven presence of one of the following common translocations by FISH were excluded: *IGH*::*BCL2*, *IGH*::*CCND1*, *IGH*::*MYC* and *IGH*::*BCL3*. The exception from the latter were two patient samples with *IGH*::*BCL3* fusion that showed evidence of additional *IGH*-translocations and two patient samples with breakpoints in 8q24 downstream (3’UTR) from *MYC*. The initial diagnosis of all patients was CLL diagnosed by local standards (flow cytometry, cell count and microscopy) or within the German CLL Study Group (GCLLSG) (*n* = 144). Tumor DNA was extracted from CD19-sorted cells from peripheral blood, lymph nodes or bone marrow (Supplementary Table 1). As controls, 20 CLL without *IG*-translocations from the Department of Internal Medicine 3 of the Ulm University Medical Center were used (Supplementary Table 2). Informed consent was obtained from all patients. Proteins from CD19-sorted peripheral blood B-cells, Jurkat and Daudi cell lines were extracted for Western blotting. Control RNA for RNA sequencing was extracted from 30 B-cell lymphoblastoid and lymphoma lines (Supplementary Table 3). Identity of the cell lines was verified by STR profiling. The study was approved by the Ethics Committee of the University Medical Center Ulm (No. 464/19).

### Fluorescence in situ hybridization (FISH)

All 144 patient samples were characterized on fixed cells extracted from peripheral blood, bone marrow or lymph nodes using interphase FISH. All samples were tested for the detection of breakpoints in *IGH*, *BCL3* [17]*, MYC, CCND1* and *BCL2* using break-apart probes (BAP) prior to the initiation of the study (Supplementary Table 4). In selected samples with available material (Supplementary Fig. 1), we verified findings and screened for translocation partners using BAP and dual color dual fusion (DCDF) probes. For detection of copy number variations (CNVs) (deletion 11q, trisomy 12, deletion 13q, deletion 17p), 142 patient samples were analyzed using commercial assays (Abbott, Chicago, IL, USA, and Metasystems, Altlussheim, Germany). FISH was performed according to standard protocols [18, 19]. For details see Supplementary Table 4.

### Targeted capture-based sequencing and whole genome sequencing (WGS)

For the identification of *IGH*-translocation junctions we followed two approaches: 50 patient samples in which DNA of sufficient amount and quality was available were subjected to custom WGS and in-house targeted capture-based sequencing was performed in ten additional samples. 44 genes were included in the targeted capture-based assay (for details see Supplementary Table 5, Supplementary Figs. 1 and 2, and Supplementary Methods). The exact positions for the class *IG*-switch regions in the *IGH* locus were extracted from Hübschmann et al. [20]. In selected cases with available material, Sanger Sequencing was performed to verify junctional segments (Supplementary Methods and Supplementary Table 6).

### Analyses of CLL candidate genes, *IGHV* mutation status and stereotyped subsets

A custom Illumina AmpliSeq library was employed to analyze DNA from 132 patient samples with *IGH*-translocated CLL, targeting 15 genes (Supplementary Methods) known to be recurrently mutated in CLL. Bioinformatic analysis followed previously described methods [21], detailed in the Supplementary Methods. The mutation frequencies were compared to published data from unselected CLL populations [21–23]. *IGHV* mutation determination and stereotyped subset analyses were performed as described recently [24, 25] and detailed in the Supplementary Methods, along with statistical analyses and visualization.

### Gene expression analysis

For RNA expression analyses the HTG EdgeSeq Pan B-Cell Lymphoma Panel (PanB panel) was performed on cell lysates from six patients with *IGH*-translocated CLL and five patients with CLL without *IG*-translocations. Custom service RNA sequencing (by BMKGENE, Münster, Germany) was performed from three patients with *IGH*::*NKX2.6*-translocated CLL and 30 B-cell lymphoblastoid and lymphoma lines (Supplementary Methods and Supplementary Table 3). Moreover, quantitative PCR (qPCR) was performed to study *NKX2.6, NKX3.1*, and *ENTPD4* transcriptional expression by the *IGH*::*NKX2.6* junction in three patients (Supplementary Methods and Supplementary Table 7). Single-cell RNA sequencing data from tonsils was extracted from published data [26] (Supplementary Methods and Supplementary Table 8).

### Western Blot analyses for NKX2.6 and BCL11A proteins

Proteins were extracted from cells of three patients with *IGH*::*NKX2.6*-translocated CLL, four patients with *IGH*::*BCL11A*-translocated CLL, five patients with CLL without *IG*-translocation, CD19-sorted normal peripheral blood B-cells, P0 mouse brain (as positive control for *BCL11A*) and the two cell lines Jurkat (as positive control for *NKX2.6*) and Daudi (as negative control for *NKX2.6*, for details see Supplementary Methods).

## Results

### Study cohort of CLLs with *IG*–translocation

CLL samples of 144 patients with breakpoints in the *IGH* loci, detected by FISH, were studied. Cases with the common translocations *IGH*::*BCL2*, *IGH*::*CCND1*, *IGH*::*MYC* and *IGH*::*BCL3*, detected by FISH, were excluded. As the focus of the study was on *IGH-*translocations, the cohort was not tested for concomitant *IGK* or *IGL* locus translocations. Of the 144 samples, 101 (70%) were from male patients. Among the 113 patient samples with clinical data from clinical trials, the median age at diagnosis was 65 (range 37-95) years (Supplementary Table 1). To estimate the overall incidence of *IGH*-translocations other than *IGH*::*CCND1* in CLL, we focused on a reference cohort of 3,832 previously untreated patients enrolled in first-line trials of the German CLL Study Group (GCLLSG). Within this cohort, in 40 patients (1%) the CLL cells harbored an *IGH*::*BCL2*-translocation, in 26 patients (0.7%) an *IGH*::*BCL3*-translocation and in 12 patients (0.3%) an *IGH*::*MYC*-translocation. In a total of 42 patients (1.1%) the CLL cells carried an *IGH-*translocation other than *IGH::CCND1, IGH::BCL2, IGH::BCL3*, or *IGH::MYC*.

### Identification of *IGH*-translocation partners and characterization of the mechanism underlying the illegitimate recombination

**Fig. 1:**
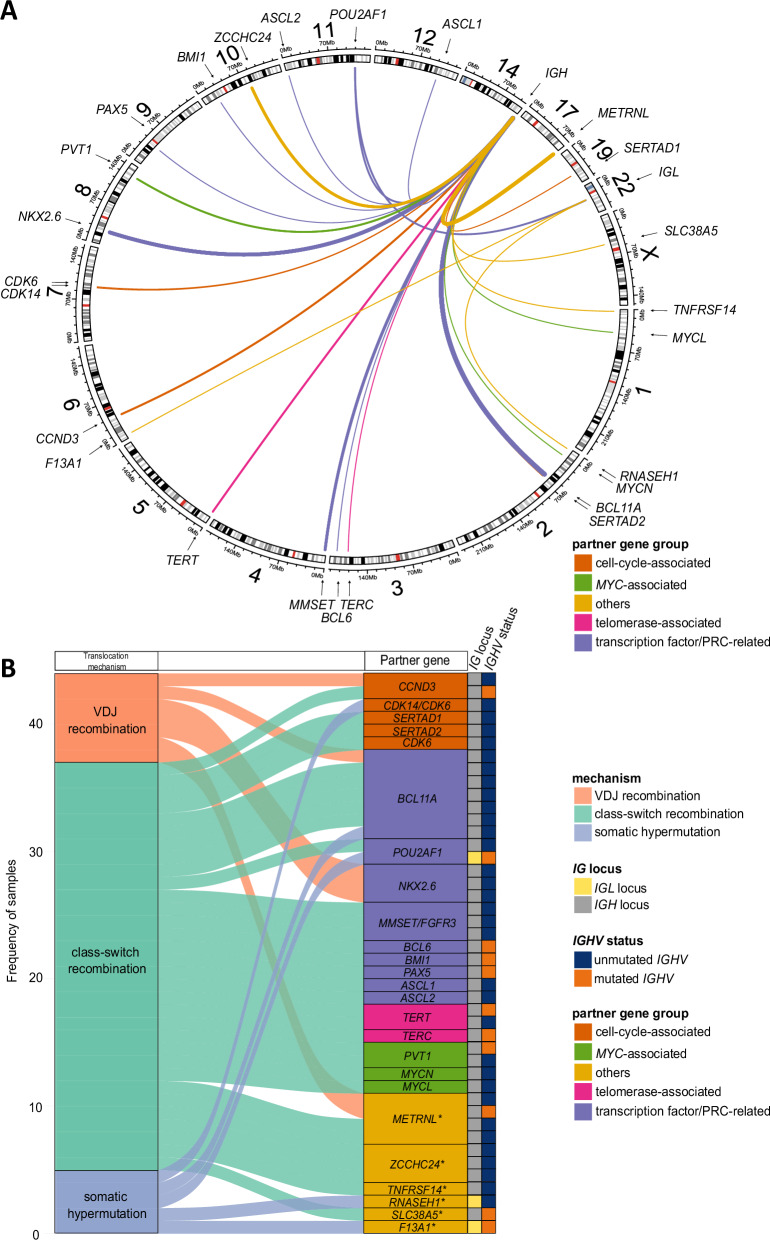
Identified translocation partners in *IG*-translocated CLL. **A** Circos plot of the 25 translocation partners identified by next generation sequencing in the 60 patient samples analyzed. The 25 genes can be separated in five different groups: cell-cycle genes, transcription factors/polycomb repressive complex (PRC) genes, telomerase-associated genes, *MYC*-associated genes, others. **B** Sankey diagram of the 25 identified translocation partners and their mechanisms underlying the illegitimate recombination. On the right side the genes are annotated by their affected *IG*-locus and their *IGHV* status. * = gene closest to the breakpoints or likely affected by the translocation.

To characterize the translocation junctions of the *IGH*-locus with the partner locus on both derivative (der) chromosomes, the der(14) and the derivative chromosome containing the partner gene, we performed WGS in 50 patients and targeted capture-based sequencing in ten patients. This analysis identified 25 translocation partners across 15 chromosomes in 38/60 samples (Fig. 1A). In 5/38 samples we identified more than one *IG*-translocation. In total, we identified 41 *IGH*-translocations and, as additional finding since *IGL* was not the primary focus, three *IGL*-translocations by NGS. *IGK*-translocations were not identified in the cases that underwent NGS. The mechanisms underlying the illegitimate *IGH-*rearrangements inferred from the junctional sequences of 41 translocations affecting the *IGH* locus were aberrant CSR (78%, 32/41) involving *IGHM* (9/41), *IGHG3* (9/41), *IGHG2* (7/41), *IGHG1* (4/41), *IGHA1* (3/41), *IGHG4* (3/41) and *IGHE* (1/41) with four translocations having breakpoints in two different switch regions. In 17% (7/41) of cases aberrant VDJ recombination, and in 5% (2/41) aberrant somatic hypermutation (SHM) was the most likely mechanism underlying the translocation (Fig. 1B). All three translocations in the *IGL* were inferred to be derived from aberrant SHM.

A total of 25 different translocation partners were involved in the *IGH* or *IGL* locus translocations (Supplementary Table 9). Nine translocation partners were identified in more than one patient sample (*BCL11A*, *METRNL, NKX2.6, MMSET, TERT, ZCCHC24, PVT1, CCND3, POU2AF1*). We additionally screened samples not undergoing sequencing by FISH (BAP and DCDF FISH probes for *MYCN*, *TERT*, *BCL6*, *CBFA2T3*, *BCL11A*, *MMSET/FGFR3*) using sequencing-verified cases as positive controls (Supplementary Results and Supplementary Fig. 1). Combining results from NGS and FISH, we detected the translocation partner in 50 *IG*-translocation events with recurrent partners being *BCL11A* (*n* = 12)*, METRNL* (*n* = 4)*, NKX2.6* (*n* = 3)*, MMSET* (*n* = 3)*, TERT* (*n* = 3)*, ZCCHC24* (*n* = 3)*, PVT1* (*n* = 2)*, CCND3* (*n* = 2) and *POU2AF1* (*n* = 2).

The translocation partners grouped into five categories based on the predicted functions of the encoded protein, namely cell-cycle related genes (*CCND3, CDK14/CDK6, SERTAD1, SERTAD2*), transcription factor/polycomb repressive complex (PRC)-related-genes (*BCL11A, POU2AF1, NKX2.6/NKX3.1, BCL6, BMI1, PAX5, ASCL1, ASCL2, MMSET*), telomerase-associated genes (*TERT* and *TERC*), *MYC*-related genes (*MYCN, MYCL, PVT1*) and other genes (*METRNL, ZCCHC24, TNFRSF14, SLC38A5, F13A1, RNASEH1*).

### *IGHV* mutations status and stereotypes in CLL with *IGH-*translocation

Analyzing the data for the *IGHV* mutation status, we detected an unmutated *IGHV* in 74% (107/144) of patient samples and a mutated *IGHV* in 24% (35/144) of patient samples. In two samples the *IGHV* mutation status could not be determined. Next, we investigated the *IGHV* mutation status in the five identified translocation groups and the remaining unknown translocation partners separately. The mutational status of *IGHV* was predominantly unmutated in most groups (cell-cycle: 80% (4/5), transcription factor/PRC: 88% (21/24), telomerase-associated: 50% (2/4), *MYC*-associated: 75% (3/4), others: 67% (5/7), remaining unknown partners: 72% (72/100)).

Next, we performed stereotype subset analysis to investigate potential enrichment of specific variable region repertoires [27]. We identified stereotype #2 in 5% (6/125), #8 in 2.4% (3/125) and #1 in 2% (2/125). Other subsets, including #3, #7, #10, #64B, #99, and #148B, were each found in 1% (1/125) of cases. Notably, when compared to the general CLL population [25], our cohort of CLL with *IGH*-translocation showed numerically higher occurrence of stereotype subset #8. Due to small sample size, no significance could be determined (Supplementary Fig. 3A).

### Group-specific analysis of copy number aberration and mutational landscape of CLL with *IGH-*translocation

We subsequently investigated genomic aberrations in the overall cohort of *IGH*-translocated CLL. The most prevalent aberrations observed were deletion 13q (41%, 58/142), trisomy 12 (29%, 41/142), *NOTCH1* mutations (20%, 26/132), *TP53* mutations (18%, 24/132), deletion 11q (16%, 23/142), deletion 17p (15%, 22/142) and *SF3B1* mutations (15%, 20/132). Significant enrichment in comparison to typical CLL [22] was detected in deletion 11q (FDR < 0.001, OR: 6), deletion 17p (FDR < 0.05, OR: 2), trisomy 12 (FDR < 0.001, OR: 2) and in mutations in *TP53* (FDR < 0.01, OR: 2)*, BIRC3* (FDR < 0.001, OR: 4), *NOTCH1* (FDR < 0.05, OR: 2), *XPO1* (FDR < 0.01, OR: 3) and *NFKBIE* (FDR < 0.001, OR: 8), and significant depletion of *POT1* (FDR < 0.02, OR: 0.06).

The group of cases with *IGH*-partners from transcription factor/PRC genes demonstrated significant enrichment of mutations in *XPO1* (FDR < 0.001, OR: 8). Significant enrichment in *FBXW7* mutations (FDR < 0.01, OR: 13) was detected in the “others” group compared to typical CLL [22].

The *IGH*-translocated CLL with remaining unknown partner gene, showed significant enrichment in deletion 11q (FDR < 0.001, OR: 7), deletion 17p (FDR < 0.01, OR: 3), trisomy 12 (FDR < 0.05, OR: 2), *TP53* (FDR < 0.001, OR: 3)*, BIRC3* (FDR < 0.05, OR: 3) and *NFKBIE* (FDR < 0.001, OR: 7) mutations compared to typical CLL [22] (Supplementary Figs. 3B, 4 and 5). For the comparison of the CLL candidate gene mutation analysis from panel sequencing and WGS data see Supplementary Results.

### Expression of potential translocation partner genes in CLL with *IGH-*translocation

This study identified 12 translocation partner genes (*SERTAD1, SERTAD2, NKX2.6, RNASEH1, ASCL2, MYCL, METRNL, ZCCHC24, CDK14, SLC38A5, TNFRSF14*, and *F13A1)* that have not been previously reported as *IGH*-partners in CLL or other B-cell neoplasms to our knowledge. We analyzed published single-cell RNA sequencing data from a human tonsil reference [26] for these potentially novel genes targeted by the translocation. This approach enabled the identification of candidate target genes with expression patterns indicative of functional relevance at specific points during B-cell maturation. Notably, *RNASEH1, METRNL, SERTAD2* and *ENTPD4* exhibited elevated transcriptional activity starting with the dark zone proliferating (DZ prolif) B-cells and going on in the germinal center (Supplementary Fig. 6). The remaining genes exhibited either no detectable expression (*F13A1, SLC38A5, ZCCHC24, B3GNTL1, ASCL2, ASCL1, NKX3.1, NKX2.6, TERC, TERT*), which in the event of a translocation could result in ectopic and aberrant activation, or relatively stable expression levels throughout B-cell-development (*TNFRSF14, SERTAD1, BCL11A*).

Furthermore, in selected patient samples we investigated whether the *IGH* enhancer hijacking led to increased expression of the named candidate oncogenes. For example, no *TERT* transcripts were detected in the single-cell RNA sequencing dataset. However, RNA expression analyses of *IGH*::*TERT*-translocated cases revealed transcriptional upregulation of *TERT* in the *IGH*::*TERT* translocations (*n* = 2) and also modestly in one case with *IGH*::*TERC* translocation (Supplementary Fig. 7A). This indicates that potential translocation partner genes can be transcriptionally activated by translocation events, even if they show no detectable RNA expression in the single cell analysis atlas during normal B-cell development. For other translocation partners, analysis was limited to single cases (Supplementary Results, Supplementary Fig. 7B–D).

### Characterization of the protein isoform expression of BCL11A in *IGH*::*BCL11A*-translocated CLL

**Fig. 2:**
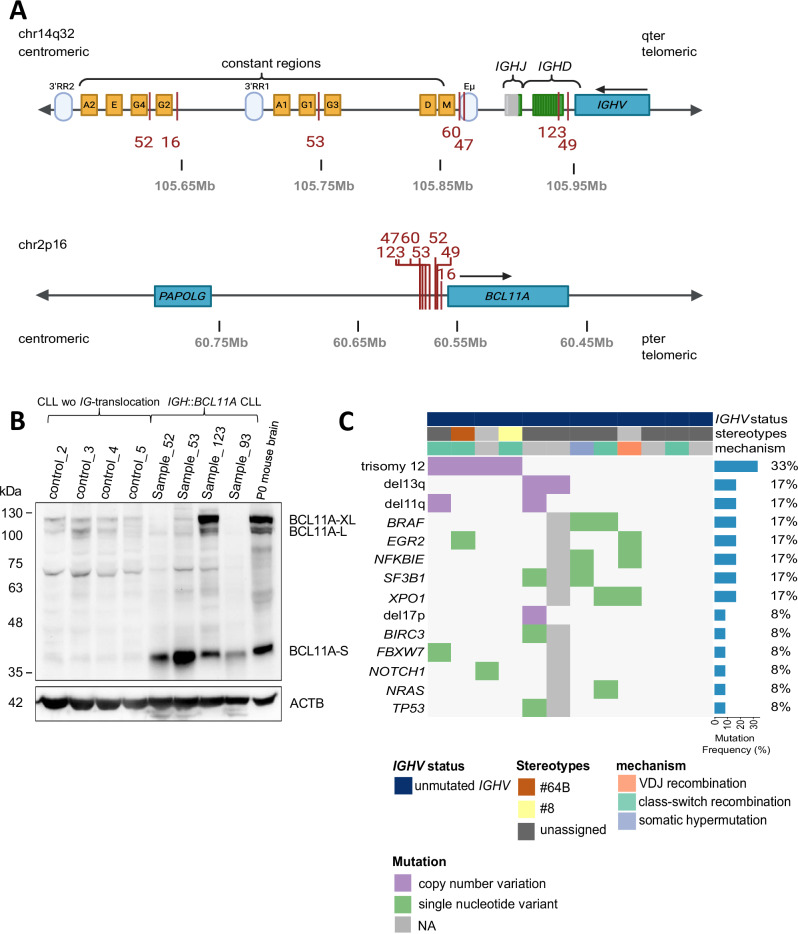
Characterization of the *IGH*::*BCL11A*-translocated CLL. **A** *IGH*-breakpoints and breakpoints in chromosome 2p16 of the seven CLL samples (22 breakpoints, one red line belongs to two breakpoints, red numbers indicate each case) with *IGH*::*BCL11A*-translocation. **B** Western Blot of the protein expression of different BCL11A transcripts (BCL11A XL, L, S) and the housekeeping gene *ACTB* for four *IGH*::*BCL11A*-translocated CLL samples, four CLL samples without *IG*-translocation and one sample of P0 mouse brain as positive control. **C** Oncoplot of recurrently mutated genes (in green) and copy number variations (in purple) in CLL samples with *IGH*::*BCL11A*-translocation. Top annotations show *IGHV* mutation status, stereotypes, and mechanism. The genetic aberrations on the y-axis are sorted according to their frequency.

Based on our NGS and FISH results, the *IGH*::*BCL11A*-translocation emerged as the most prevalent translocation in our cohort, occurring in twelve cases. Junctional sequence analysis, available from 7/12 cases, suggested the underlying translocation mechanism to be aberrant CSR in five cases, aberrant VDJ recombination in one case and aberrant SHM also in one case (for details see Fig. 2A and Supplementary Results). To assess whether this translocation leads to overexpression of BCL11A-protein and to determine which protein isoforms are produced, we performed immunoblotting for three BCL11A isoforms (BCL11A-XL, L, and S) from four *IGH*::*BCL11A*-translocated CLL samples with available material, four CLL samples without *IG*-translocation and one respective control. Strong expression of BCL11A-S was detected consistently in all four *IGH*::*BCL11A*-translocated CLL samples, with additional expression of BCL11A-XL and BCL11A-L observed in one case. BCL11A expression was only minimally detectable in the four CLL samples without *IG*-translocation (Fig. 2B). These findings suggest that *IGH*::*BCL11A*-translocations are associated with marked overexpression of mainly the BCL11A-S isoform. *IGHV* was consistently unmutated whereas a highly heterogeneous distribution of CNVs and mutations in recurrently mutated genes was observed in the twelve *IGH*::*BCL11A*-translocated CLL samples (trisomy 12 was present in four cases, for details see Fig. 2C and Supplementary Results).

### Characterization of the *IGH*::*NKX2.6*-translocation in CLL

**Fig. 3:**
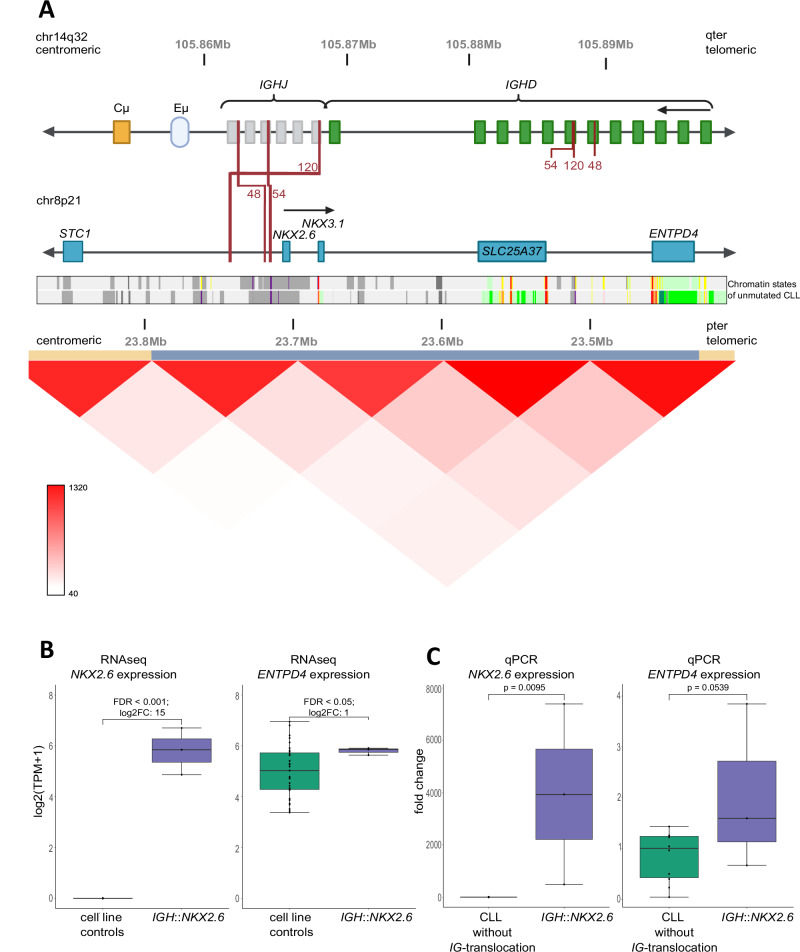
Characterization of the *IGH*::*NKX2.6*-translocated CLL. **A** *IGH*-breakpoints and breakpoints in chromosome 8p21 of the three CLL samples (twelve breakpoints, in chromosome 8 one red line belongs to two breakpoints, red numbers indicate each case) with *IGH*::*NKX2.6*-translocation and the chromatin states of two samples with unmutated CLL according to Beekman et al. [68]: yellow: weak enhancer, orange: strong enhancer, red: promotor, light green: weak transcription, dark green: strong transcription, purple: poised promotor, light gray: heterochromatin, dark gray: Polycomb repressive. The *IGH* constant regions were omitted due to the absence of breakpoints. The breakpoints in *IGHD* belong to the derivative chromosome 8. The HiC data for human blood data on chr8:23400000-23900000 (hg38) was extracted from https://3dgenome.fsm.northwestern.edu/view.php. **B** Boxplots from RNA sequencing data of *IGH*::*NKX2.6*-translocated CLL (*n* = 3) and control cell lines (BL, HL, DLBCL, HGBCL-11q, PMBCL, LCL, NLPHL, *n* = 30) on x-axis and log2(TPM + 1) on the y-axis showing the significant upregulation of *NKX2.6* and *ENTPD4* expression. **C** Boxplots from qPCR data of *IGH*::*NKX2.6*-translocated CLL (*n* = 3) and CLL without *IG*-translocation (*n* = 10) on x-axis and fold change on the y-axis showing the upregulation of *NKX2.6* and *ENTPD4* expression.

A particularly intriguing finding was the identification of a recurrent translocation between a region of only 30 kb in 8p21.2 and the *IGH* locus mediated by aberrant VDJ recombination. In total, we identified three patients with CLL with 12 breakpoints for the derivative chromosomes and in addition a follow-up sample from sample 48, four years after the initial sample (data not shown). The fusions were verified by PCR-based Sanger sequencing in all cases. All breakpoints on chromosome 8 were located centromeric to *NKX2.6* (10 to 40 kb upstream of putative transcriptional start site). The centromeric breakpoints in chromosome 14 were located in the *IGHJ1/IGHJ4/IGHJ6*-segments and the telomeric in *IGHD3-22*- and *IGHD2-21*-segments. N-nucleotides (10 to 23 bp) and recombination signal sequences (RSS)-sites (RSS12 and RSS23) were identified in proximity (1 to 12 bp away) to the breakpoints. The presence of these characteristic elements (N-nucleotides and RSS) in conjunction with the specific location of the breakpoints within the *IGH* locus, strongly indicates that VDJ recombination was the underlying mechanism responsible for generating these illegitimate rearrangements. Based on the location of the breakpoints and predicted underlying mechanism acting all enhancer segments of the *IGH* locus are supposed to be retained on chromosome 14 in line with activation of the target oncogene from chromosome 8 and the derivative chromosome der(14)t(8;14)(p21.2;q32). Thus, a candidate oncogene would be supposed to be located telomeric of the breakpoints in the short arm of the chromosome 8. We performed RNA sequencing based gene expression analysis and qPCR in all three CLL samples with *IGH*::*NKX2.6*-translocation to see whether the genes telomeric of the breakpoint cluster (in particular *NKX2.6, NKX3.1, SLC25A37* and *ENTPD4* located in the same topologically associated domain, see Fig. 3A) were deregulated by the translocation (Fig. 3B, C). We identified a significant upregulation of *NKX2.6* (RNAseq: FDR < 0.001, logFC: 15, qPCR: *p* < 0.01) and *ENTPD4* (FDR < 0.05, logFC: 1, qPCR: *p* < 0.01) in the *IGH*::*NKX2.6*-translocated CLL, but no upregulation of *NKX3.1* and *SLC25A37* for the RNA sequencing data compared to cell line controls and only significant upregulation of *NKX2.6* (*p* < 0.01) with the qPCR data compared to CLL without *IG*-translocation. This suggests a potential role for *NKX2.6* in CLL pathogenesis, which is moreover the protein-coding gene mapping closest to the breakpoint and, thus, to the *IGH* sequences after translocation. *IGH*::*NKX2.6* fusion transcripts were identified in the *IGH*::*NKX2.6*-translocated CLL but not in the control cell lines and published CLL [28] by RNA sequencing. *TDT*, *RAG1* and *RAG2* transcripts were absent in the *IGH*::*NKX2.6*-translocated CLL (Supplementary Fig. 8). No NKX2.6 or NKX3.1 protein expression was detectable using NKX2.6 polyclonal (Invitrogen, Waltham, MA, USA) and Anti-NKX3.1 (Abcam, Cambridge, UK) antibodies in the three *IGH*::*NKX2.6*-translocated CLL (Supplementary Fig. 9A). ENTPD4 showed higher protein expression in the *IGH*::*NKX2.6*-translocated CLL in comparison to one CLL sample without *IG*-translocation. The CNVs and somatic mutations showed a very heterogenous distribution in the samples with deletion 13q present in 2/3 patients (Supplementary Fig. 9B), whereas all three patient samples were characterized by an unmutated *IGHV* status.

### *METRNL* as potential translocation partner gene in CLL with *IGH-*translocation

Another recurrently identified partner gene, not identified in CLL or other B-cell malignancies before, was *METRNL*. It was detected in four CLL samples with *IGH*-translocation and breakpoints in chromosome 17q25.3. In three of these cases, the breakpoints on chromosome 17q were located centromeric to *METRNL* within *B3GNTL1*, predicted to result in a disrupted, non-functional *B3GNTL1* protein due to the translocation. Two cases were caused by aberrant VDJ recombination and one case was caused by aberrant CSR. The breakpoint in the remaining case was located telomeric to *METRNL* and was caused by aberrant CSR (Supplementary Fig. 10). Thus, *METRNL* is proposed as the oncogenic gene affected by the translocation. Investigation of *METRNL* expression in a single case showed upregulation of *METRNL* (Supplementary Fig. 7E). Remarkably, analysis of published single-cell RNA sequencing data [26] from tonsils demonstrated that *METRNL*, but not *B3GNTL1*, to be detectable at low levels in germinal center B-cells and plasma cells (Supplementary Fig. 6). These findings suggest that *METRNL* may have a more significant role than *B3GNTL1* in B-cell development.

## Discussion

Here we characterized a large cohort of 144 patients with CLL with *IGH*-translocation for *IGH*-translocation partner genes, mechanism involved in the generation of the translocation, *IGHV* mutational status, chromosomal imbalances, somatic mutations and expression of selected genes. In a reference cohort of 3,832 previously untreated patients with CLL from first-line GCLLSG trials, *IGH*::*BCL2*, *IGH*::*BCL3* and *IGH*::*MYC* were observed in the CLL cells of 1%, 0.7% and 0.3% of patients, respectively, consistent with frequencies reported in the literature [1, 14, 15, 29–31]. *IGH*-translocations other than *IGH::CCND1, IGH::BCL2, IGH::BCL3* or *IGH::MYC* were observed in 1.1% of cases, a prevalence aligning with previously published data [1].

We identified 25 *IGH*-translocation partners and showed the translocations to be mainly caused by aberrant CSR with predominant involvement of *IGHG* and *IGHM* genes. The majority of *IGH*-translocated CLL samples in our study carried an unmutated *IGHV* (*n* = 107/144, 74%) consistent with the 72% reported in the literature [16]. These findings suggest that the tumor cells in the majority of these CLL cases likely have not undergone SHM in germinal centers. Remarkably, in 2/32 (6%) CLL with unmutated *IGHV* the *IGH* breakpoints showed evidence for aberrant SHM as mechanism of the *IGH*-translocation, suggesting that germinal center passages of the tumor cells are not fully excluded in *IGHV* unmutated CLL. Aberrant CSR was the most common mechanism causing translocation breakpoints in *IGHV* unmutated CLL (*IGH*-translocation caused by CSR with unmutated *IGHV* in our cohort: *n* = 24/32, 75%) followed by aberrant VDJ recombination (*n* = 6/32 *IGHV* unmutated cases; 19%). The aberrant CSR mediated translocations in these cases might possibly be driven either by T-cell dependent class-switch outside the germinal center, only shown yet in murine models [32], or T-cell-independent immune responses [33, 34]. In summary, these findings might indicate development of these *IGHV* unmutated CLL with *IGH*-translocations from B cells at an earlier stage outside the germinal center, also supported by others [1, 35]. Remarkably, no cases of CLL with mutated *IGHV* and with *IGH*-translocation caused by aberrant SHM were identified in our cohort. However, as outlined above, six cases with *IGH*-translocation caused by aberrant VDJ recombination and with unmutated *IGHV* were identified suggesting the translocation to occur at an early stage of B-cell development. Contrary, in CLL samples with mutated *IGHV* all translocations were associated with aberrant VDJ recombination (*n *= 1/9, 11%) or CSR (*n* = 8/9, 89%) suggesting that the translocations occurred before or at least outside the germinal center and independent of the SHM process. This finding might disentangle the developmental state of the cell undergoing transformation and the normal counterpart cell and might be in line with the hypothesis, that CLL with mutated *IGHV* and absence of *IGH*-translocation might resemble features of memory B cells [35–38].

Among the 25 identified translocation partners, seven were previously known in CLL (*TERT, MMSET/FGFR3, ASCL1, BMI, BCL11A, CDK6, PAX5*) [2, 11, 33, 39–48], while others such as *CCND3*, *BCL6, PVT1, POU2AF1, TERC* and have been reported in related B-cell malignancies [49–57]. Notably, we identified 12 translocation partners (*SERTAD1, SERTAD2, NKX2.6, RNASEH1, ASCL2, MYCL, METRNL, ZCCHC24, CDK14, SLC38A5, TNFRSF14* and *F13A1*) to the best of our knowledge not previously reported with CLL or other B-cell malignancies.

The most recurrent translocation in our cohort was the *IGH*::*BCL11A*-translocation identified in twelve patient samples with the protein isoform BCL11A-S expressed in four cases. *BCL11A* (chr2p16), a member of the zinc-finger transcription factor family and transcriptional repressor [58, 59], exists in four different isoforms (BCL11A-XL, -L, -S and -XS), which differ in their length and the presence or absence of *C2H2* zinc finger domains [59]. The *IGH*::*BCL11A*-translocation is a recurrent event in CLL [33, 60, 61]. Previous studies have reported that translocation breakpoints occur in *IGHG2* and that this translocation is associated with unmutated *IGHV* [33, 62]. Furthermore, it has been shown that CLL cases harboring the *IGH*::*BCL11A*-translocation express the three most common BCL11A isoforms: XL, L and S [61]. These three isoforms contained the first three exons. BCL11A-XL contains the fourth exon as well, whereas BCL11A-L and S show alternative splicing of exon 4 to exon 5 resulting in two differently spliced isoforms [59]. BCL11A-XL is predominantly detected in normal B-cell populations [61] and acts as the mainly expressed transcript with the main function as transcriptional repressor [61, 63]. In contrast, BCL11A-S is primarily present in malignant B-cell lines [61]. These prior observations are consistent with our findings, as BCL11A-S emerged as the main isoform expressed in *IGH*::*BCL11A*-translocated CLL cases within our cohort. BCL11A-S, the shorter isoform, lacks the critical domain for the full repressor function [59, 61]. Therefore, the BCL11A-S isoform is believed to contribute to cancer pathogenesis by its inability to regulate target genes [61, 64].

We identified a series of previously undescribed *IGH*-translocation partners, with recurrence of *NKX2.6* (chr8p21) involved in aberrant VDJ-recombination mediated *IGH* enhancer hijacking in three cases of *IGHV* unmutated CLL. *NKX2.6*, encoding a homeobox transcription factor primarily known for its role in heart development [65], has not been previously implicated in CLL pathogenesis. In mice it was already shown that Nkx2.6 has an expressional behavior similar to that of Nkx2.5 in the pharynx and heart, and to Nkx2.3 in the pharynx [65]. *NKX2.5* has been identified as a translocation partner in T-cell acute lymphoblastic leukemia (ALL) [66], suggesting its potential importance in lymphoid neoplasms. Furthermore, *NKX2.3* is overexpressed in MZL [67]. In contrast to the typical silent promoter and lack of RNA expression of *NKX2.6* in the normal CLL populations [68], our RNA sequencing and qPCR data demonstrated clear upregulation of *NKX2.6* in *IGH*::*NKX2.6* translocated CLL, suggesting a functional consequence of the translocation. Although NKX2.6 and the neighboring NKX3.1 proteins were not detected by immunoblotting, we hypothesize this may reflect the presence of a potential fusion protein involving NKX2.6 and the *IGH* locus due to fusion transcripts or structural alterations resulting from the translocation that affect protein stability or detection. Additionally, genomic variants detected in *NKX2.6* across all three cases (Supplementary Table 10) may influence protein detection, as the used antibody recognizes amino acids 224 to 274 of NKX2.6. Notably, one variant (hg38: chr8:23702632) detected in sample 54 was located within this region. This finding further highlights the biological complexity and may contribute to the observed absence of protein expression. The unmutated *IGHV* status and VDJ recombination-mediated breakpoints in all three patient samples point to an early event in B-cell development, likely occurring in bone marrow area. This hypothesis is supported by the findings that *NKX*2.5, related to *NKX2.6*, was often associated with T-ALL [66, 69], a disease hypothesized to be originating from T-cell precursor cells [70]. In contrast, CLL may originate from mature CD5 + B-cells or CD5 + CD27+ post-germinal center B-cells [71]. However, it was also found that hematopoietic stem cells generate B-cells with CLL-like phenotypes, suggesting that aberrant hematopoietic stem cells may represent early pre-leukemic precursors that can evolve into CLL following the acquisition of additional genetic alterations [72, 73]. Therefore, our findings open questions on the potential cell of origin in B-cells at least in a subset of CLL.

An additional translocation partner, not previously described in CLL pathogenesis, was *METRNL* (chr17q25), identified as a recurrent translocation partner gene in four cases. *METRNL* encodes the meteorin like glial cell differentiation regulator which is secreted in the tumor microenvironment [74] suggesting a potential role in tumor biology. Our WGS data indicated that *METRNL* is the most plausible candidate driver gene within this locus, as the observed breakpoints disrupted the coding sequence of *B3GNTL1*, located centromeric to *METRNL*, while no protein-coding genes are present on the telomeric side (see http://genome.ucsc.edu for hg38). Transcriptomic analyses revealed *METRNL* overexpression in one case harboring an *IGH*::*METRNL*-translocation, and single-cell RNA sequencing further demonstrated its expression in germinal center B-cells. Together, these findings raise the possibility that *METRNL* represents a biologically relevant target gene in *IGH*::*METRNL*-translocated CLL.

In conclusion, our study identifies new *IGH*-fusion partners of potential pathogenic significance and defines CLL with *IGH*-translocations as a distinct molecular subgroup with unique genetic features and potentially different cellular origins. The high frequency of unmutated *IGHV* coupled with evidence of aberrant CSR suggests a complex interplay between developmental stage and oncogenic events. The identification of novel translocation partners, particularly *NKX2.6* and *METRNL*, shows new options for investigating CLL pathogenesis and may lead to refined prognostic markers and therapeutic targets.

## Supplementary information

Supplementary Tables

Supplementary Methods and Results

Supplementary Figures

## Data Availability

Whole Genome Sequencing data and targeted breakpoint NGS data are available upon request from the corresponding author.
