# Supplementary material for: The spectrum of immunoglobulin heavy chain enhancer hijacking in chronic lymphocytic leukemia
Source: Leukemia. 2026 Apr 23;40(7):1401–10. doi: 10.1038/s41375-026-02902-9 (PMC13323050; doi:10.1038/s41375-026-02902-9)
Supplement: Supplementary file 2 — Supplementary Methods and Results [file 41375_2026_2902_MOESM2_ESM.docx]

**Supplementary Information**

**Supplementary Methods**

**Targeted capture-based and Whole Genome Sequencing (WGS)**

For the identification of *IGH*-translocation junctions we followed two approaches: 50 patient samples in which DNA of sufficient amount and quality was available were subjected to custom whole genome sequencing (WGS), using TruSeq PCR-free Library Preparation (350bp) and NovaSeq X Plus sequencing by a service provider (Novogene, Martinsried, Germany). The median of the mean coverages per sample in the whole genome samples was 34x (range: 29x to 51x). In addition, in-house targeted capture-based sequencing was performed in ten additional samples using the SureSelectXT HS Target Enrichment System (2x150bp) and sequenced in a NextSeq 550 (Illumina, San Diego, CA, USA) in-house. The median of the mean coverages per sample in the targeted capture-based approach was 113x (range: 55x to 504x). The depth of coverage for each sample was determined using samtools (version 1.19.2) (1). For WGS data, the mean coverage per sample was calculated by considering only the average depth of chromosomes 1 through 22. In the case of the targeted capture-based approach, the mean coverage for each sample was computed by focusing solely on the average depth of the *IGH*-locus (hg38: chr14: 105479370-106470468) as it was the only gene affected in all cases. R software (version 4.3.1) was employed to calculate the overall mean and median of the mean coverages across all samples. The alignment of the processed fastq files to the human reference genome (GRCh38) was accomplished using the BWA-MEM algorithm (version 2.2.1) (2). Subsequently, samtools (version 1.19.2) (1) was utilized to generate, sort, and index the BAM files. FastQC (version 0.12.1) (3) was employed for quality control purposes. To identify immunoglobulin translocations, Igcaller (version 1.3) (4) was used, followed by visual verification with the Integrative Genomic Viewer (IGV, version 2.17.0) (5). Somatic variant calling for the WGS was performed using the GATK workflows (https://github.com/gatk-workflows, viewed at 11.04.25).

**Analysis of breakpoint junctions at *IGH* gene locus of the translocated allele and verification by Sanger Sequencing**

The junctional sequences of the *IGH* loci with the respective partner gene were extracted from WGS and targeted sequencing data. We inspected the breakpoints with Human BLAT Search from UCSC Genome Browser (https://genome.ucsc.edu/cgi-bin/hgBlat, viewed at 09.12.24) identifying N-nucleotides at the breakpoint junctions and used the RSS database CNR-ITB for the analysis (https://www.itb.cnr.it/rss/index.html, viewed at 22.06.24) to detect the presence of cryptic restriction start sites (RSS) within 1kb up- and downstream of the breakpoints in chromosome 14 and the partner gene. The system determines if genetic sites are functional based on RIC scores, following Cowell's research (6–8). For RSS-23 sites, a score of -58.45 or higher means the site is functional (passes). For RSS-12 sites, a score of -38.81 or higher indicates functionality (passes). Scores below these thresholds mean the sites are non-functional (fail) (6–8).

**Breakpoint verification by PCR and subsequent Sanger Sequencing**

The breakpoints of 25 patient samples with *IGH* rearrangements and their partner genes (details provided in Supplementary Table 5), as identified through WGS or targeted capture-based sequencing, were validated using PCR-based Sanger sequencing. Primers were designed to target regions located 200–400 bp from the breakpoints. The PCR primers and conditions used for Sanger sequencing are listed in Supplementary Table 6. Sequencing of the PCR products was performed using the Big Dye Terminator v3.1 Cycle Sequencing Kit (Applied Biosystems, Waltham, MA, USA), and the resulting sequences were analyzed on a 3130xl Genetic Analyzer (Applied Biosystems, Waltham, MA, USA).

**CLL candidate gene mutation analysis**

To identify recurrently mutated candidate genes, we investigated available DNA from 132 patients with *IGH-*translocated CLL using a custom Illumina AmpliSeq library covering *NOTCH1, SF3B1, ATM, TP53, RPS15, BIRC3, MYD88, FBXW7, POT1, XPO1, NFKBIE, EGR2, BRAF, NRAS* and *KRAS* either for the full gene or the most commonly affected exons (9,10). Adjacent 5 bp in the intron were included to cover splice site mutations. Tumor only sequencing was performed on an Illumina MiSeq™ in 48 sample batches with the 600-cycle MiSeq Reagent Kit v3. For alignment, variant calling and annotation we used a custom bioinformatics pipeline including BWA-MEM (version 2.2.1) (2) and samtools for alignment (version 1.19.2) (1), and Varscan2 for variant calling and annotation (11) and Scandel for deletion calling. Current databases (COSMIC, 1000G, dbSNP150, ClinVar, Seshat) were taken into consideration to evaluate and report variants above a threshold of 5% mean variant allele fraction (VAF) as pathogenic/non pathogenic. Only variants classified as pathogenic or likely pathogenic were considered as mutations, while single nucleotide polymorphisms (SNPs) with frequency > 1% in healthy population, variants classified as benign or likely benign or variants of unknown significance were not reported. The frequency of mutations was compared with data of unselected CLL populations (9,10,12).

***IGHV* mutation and stereotype subset analyses**

*IGHV* mutation status was obtained from patient samples with available material (n = 142). It was assessed by amplifying the predominant V(D)J rearrangement of the heavy chain using multiplex PCR according to literature (13). The sequence of the tumor sample was compared to the germline sequence with highest homology. The *IGHV* mutation status was calculated as the ratio of the number of matching nucleotides in the *HV* gene and the total number of nucleotides in the *IGHV* germline gene. An unmutated *IGHV* mutation status was defined by an identity to the germline of 98% or higher. *IGHV* clonal sequence analysis was implemented as a standard procedure beginning in 2021. Consequently, we were able to examine 125 patient samples for their stereotype subset classification. Stereotype analysis was performed by amplification of the *IGHV*-*IGHD*-*IGHJ* gene rearrangements and subsequent Sanger sequencing to determine to the common sequence. By uploading the sequence to ARResT (https://bat.infspire.org/arrest/assignsubsets/) an assignment to one of nineteen major CLL stereotyped subsets according to relative scores and confidence assignment is performed (14). The CLL with *IGH*-translocation were compared with publicly available data for stereotypes #1, #2, #4 and #8 of early and advanced stage CLL (14).

**Reverse transcription and quantitative polymerase chain reaction (qPCR)**

Patient samples of three *IGH*::*NKX2.6*-translocated CLL and ten CLL without *IG*-translocation (as controls) were used for qPCR-based measurement of *NKX2.6, NKX3.1* and *ENTPD4* transcriptional levels. cDNA synthesis was performed using GoScript™ Reverse Transcription System (Promega, Madison, WI, USA). In brief, RNA was isolated from patient peripheral blood mononuclear cells (PBMCs) using Qiagen RNeasy mini kit. 400 ng of RNA was reverse transcribed using random hexamers according to the manufacturer’s instructions. The synthesized cDNA mix was diluted 1:10 v/v. For qPCR, the iTaq Universal SYBR Green Supermix (Bio-Rad, Hercules, CA, USA) was used. PCR reactions were set up on MicroAmp optical 384 well plates (Applied Biosystems, Waltham, MA, USA) in a total volume of 10 µl per well. Primer sequences for housekeeping genes *Actin B* and *RPL19* and the target genes *NKX2.6, NKX3.1* and *ENTPD4* are mentioned in Supplementary Table 6. Final concentration of each primer used for qPCR was 500 nM. qPCR was performed in a QuantStudio 5 qPCR-system (Applied Biosystems, Waltham, MA, USA). PCR amplification was performed for 40 cycles using denaturation at 95°C for 15 seconds followed by a one-step annealing and elongation at 60°C for 1 min as recommended in the product’s specifications.

**Gene expression analysis**

The HTG EdgeSeq Pan B-Cell Lymphoma Panel (PanB panel) consisting of 298 genes was performed according to the manufacturer’s instructions (HTG Molecular Diagnostics, Tucson, AZ, USA). To this end, cell lysates were obtained from six *IGH*-translocated CLL (one *IGH*::*PVT1*, one *IGH*::*TERC*, two *IGH*::*TERT*, one *IGH*::*MMSET*, one *IGH*::*METRNL*) and five CLL without *IG*-translocations as controls. The raw data obtained from the HTG EdgeSeq parser software was subjected to quantile normalization, with the 0.99th and 0.01th percentiles serving as the trimming points. Significance was not calculated due to the low number of samples for each group.

Custom service RNA sequencing (by BMKGENE, Münster, Germany) was performed from three patients with *IGH*::*NKX2.6*-translocated CLL and 30 B-cell lymphoblastoid and lymphoma lines (see Supplementary Table 3). For library preparation, the Hieff NGS Ultima Dual-mode mRNA Library Prep Kit for Illumina (Yeasen, Shanghai, China) was used and the qualified library was sequenced on the Illumina NovaSeq X (Illumina, San Diego, CA, USA) using paired-end 150 bp (PE150) mode. The median of the mean coverages per sample was 4298x (range: 270x to 6208x). Fastq files were trimmed using trim galore, alignment was performed using STAR (version: 2.7.10a) (15) and StringTie (v2.1.1) (16) was used to calculate gene expression values. Additional fastq files from published B-cell acute lymphocytic leukemia (B-ALLs) (17) and CLLs (18) were normalized as well. Differential gene expression of CLL with *IGH*::*NKX2.6*-translocation compared to control cell lines was determined using Deseq2 (19). Fusion transcripts were called with arriba version 2.4.0 (20). Moreover, quantitative PCR (qPCR) was performed to study *NKX2.6, NKX3.1* and *ETPD4* transcriptional expression by the *IGH*::*NKX2.6* junction in three patients (see Supplementary Table 7). The HiC data for human blood data on chr8:23400000-23900000 (hg38) was extracted from https://3dgenome.fsm.northwestern.edu/view.php.

**Single-cell RNA-seq analysis of human tonsil reference**

Single-cell RNA-seq (scRNA-seq) analysis was performed using R version 4.3.3 (21) and the Seurat package (v5.3.0) (22). We utilized the publicly available Seurat object of human tonsil cells and accompanying cell annotations from Massoni-Badosa et. al (2024) (23). To enhance the quality of the reference dataset and reduce batch effects, we included only cells derived from fresh tissue and excluded those preserved through freezing. Moreover, cells predicted to be doublets were filtered out. To conduct a focused analysis of B cells, we narrowed the tonsil dataset to include only naïve/memory B cells (NBCs and MBCs) and germinal center B cells (GCBCs). Both datasets underwent log-normalization, variable feature selection, and data scaling using Seurat with default parameters. Re-clustering of the B-cell subset was performed by merging related cell types into broader categories: NBCs, dark zone (DZ) proliferative and non-proliferative cells, light zone (LZ) cells, Reentry cells, GC-committed NBCs (types 1 and 2), MBCs, germinal-center MBCs (gcMBCs), and plasma cells (PCs). Cells annotated as “GC DZ Nonproli” were excluded from subsequent analyses. The specific mapping of original cell types to these clusters is detailed in Supplementary Table 8. Gene expression analysis was conducted using Seurat’s DotPlot() function on the respective single-cell objects.

**Western blot analyses for NKX2.6**

Proteins were extracted from cells using the RIPA/PIC solution. 40 μg of each protein sample was diluted in DTT and H2O/ per well and incubated at 70°C for 10 min. Afterwards they were centrifuged shortly to collect the liquid at the bottom of the tube. NuPAGE Bis-Tris gels (Thermo Fisher Scientific, Waltham, MA, USA) were inserted into the Wet Blot gel chamber (Thermo Fisher Scientific, Waltham, MA, USA), which was filled with 1x MOPS buffer (Thermo Fisher Scientific, Waltham, MA, USA). For the protein size of 40kDa and smaller, a NuPAGE 4-12% gel was used. The protein samples were loaded and the separation was performed at 170 V for 1h. Proteins were blotted onto a PVDF-membrane (Amersham, UK) using a tank blot cell transfer device according to manufacturer’s instructions. Prior to blotting, the PVDF-membrane was activated using absolute methyl alcohol for 30 seconds, afterwards, it was equilibrated together with filter papers (Thermo Fisher, Waltham, MA, USA) in the 1xNuPage transfer buffer (invitrogen, Waltham, MA, USA). Blotting was performed at 20 V for 1 h. To monitor a successful transfer, the membrane was stained with Novex Reversible Membrane Protein Stain (invitrogen, Waltham, MA, USA). For the staining, the membrane was incubated with Ponceau Red Staining Solution according to manufacturer´s instructions. Afterwards, the membrane was washed with H_2_O until the protein bands were clearly visible. Subsequently, the membrane was washed with H_2_O until the staining came off. The membrane was incubated in blocking solution (5% milk powder in TBST) for at least 1h at room temperature. The specific antibody against the protein of interest was diluted in 5% milk powder (1:1000). The antibody solution was added to the membrane and incubated at 4°C on a tube roller mixer overnight. The membrane was washed with TBST three times for 15 min at room temperature. The secondary antibody was diluted in 5% milk powder (1:5000) and added to the blot. Incubation was performed for 1 h at room temperature on a tube roller mixer. The membrane was washed twice with TBST for 10 min each at room temperature and then in TBS for 10 min at room temperature. SuperSignal West Dura Extended Duration Substrate (Thermo Fisher, Waltham, MA, USA) was added to the membrane for 5 min. The liquid was drawn off until the membrane ran dry. The membrane was developed using the Fusion FX (Vilber, Eberhardzell, Germany). The membrane was shortly washed with TBST Afterwards, it was incubated with Restore PLUS Western Blot Stripping buffer (Thermo Fisher, Waltham, MA, USA) at room temperature for 5 to 15 min. The membrane was washed with TBST and an additional incubation with a first antibody. The commercial NKX2.6 Human Recombinant Protein (BioCat, Heidelberg, Germany) was added as positive control for the NKX2.6 expression. The following antibodies were used: HSP90 (Cell signaling, Danvers, MA, USA, Lot-No: 6), TBP (Abcam, Cambridge, UK, Lot-No: 1033848-4), NKX2.6 polyclonal (Invitrogen, Waltham, MA, USA, Lot-No: ZG4410056), Anti-NKX3.1 (Abcam, Cambridge, UK, Lot-No: 1022506-1) and ENTPD4 (Proteintech, Rosemont, IL, USA, Lot-No: 00011598). All antibodies were validated for their sizes by cell line controls.

**Western blot analyses for BCL11A**

Total proteins were extracted from human cells and mouse brain tissue with ice-cold RIPA buffer (50 mM Tris-HCl pH 7.5, 150 mM NaCl, 0.5% Na-deoxycholate, 1% Triton X-100, 0.1% SDS) supplemented with 1x protease inhibitor cocktail (Roche) by incubating for 10 min at 4°C with gentle rotation. Brain tissue was sonicated on high power for 10 cycles (ON time: 30 seconds, OFF time: 30 seconds) using a Bioruptor Plus (Diagenode, Liège, Belgien). After removal of cell debris by centrifugation, protein concentration was measured using Bradford Plus reagent (Thermo Fisher, Waltham, MA, USA) with bovine serum albumin as standard. 2x loading buffer (120 mM Tris-HCl pH 6.8, 4% SDS, 0.02% bromophenol blue, 20 mM DTT) was added to 40 µg of protein and boiled for 7 min at 95°C. SDS-PAGE was performed with 9% polyacrylamide gel, and proteins were transferred onto a PVDF membrane (Amersham, UK). After incubation in blocking solution (5% non-fat milk (Bio-Rad, Hercules, CA, USA) in 1x TBST) for 60 min at 22-25°C, the membrane was incubated with rabbit anti-BCL11A antibody (Bethyl, Boston, MA, USA A700-073) diluted 1:1000 in blocking solution overnight at 4°C. The membrane was washed with 1x TBST several times and incubated with horseradish peroxidase-conjugated secondary anti-rabbit IgG antibody (Jackson ImmunoResearch, Ely, UK) diluted 1:2000 in blocking solution for 60 min at 22-25°C. Target protein was developed using ECL Plus western blotting detection reagents (Thermo Fisher, Waltham, MA, USA) according to the manufacturer’s conditions. After incubation in stripping buffer (200 mM glycine, 0.1% SDS, 1% Tween 20, pH 2.2) for 15 min at 22-25°C, the membrane was blocked and incubated with mouse anti-β-actin (Abcam, Cambridge, UK, Cat# ab8226) diluted 1:2000 in blocking solution overnight at 4°C followed by treatment with horseradish peroxidase-conjugated secondary anti-mouse IgG antibody and ECL Plus western blotting detection reagents. The following antibodies were used: ACTB (Abcam, Cambridge, UK, Cat # ab8226) and “pan-BCL11A“ antibody (immunogen, Waltham, MA, USA, Cat.-# A700-073) (24,25). The antibody was proven for the three different isoform expressions by cell line controls with specific antibodies (data not shown).

**Statistical analyses and visualization**

Statistical analyses were conducted using R (version 4.3.1). Continuous variables were compared using the Wilcoxon rank sum test for independent groups. All hypothesis tests were two-sided unless otherwise specified. For categorical variables, Fisher's exact test (sample size < 5) or Chi-Square test (sample size > 5) were applied. P-values were adjusted using the Benjamini–Hochberg procedure to control the false discovery rate at 5% (q<0.05). BioRender was used to generate Figure 2A, 3A, Supplementary Figure 2 and Supplementary Figure 10.

**Supplementary Results**

**Screening of partially overlapping samples with FISH**

The screening of partially overlapping samples with FISH (see Supplementary Figure 1) verified the partners in *IGH*::*BCL11A* (n = 5), *IGH*::*MMSET* (n = 3), *IGH*::*NKX2.6* (n = 3), *IGH*::*TERT* (n = 2), *IGH*::*POU2AF1* (n = 1), *IGL*::*POU2AF1* (n = 1), *IGH*::*MYCN* (n = 1), *IGH*::*BCL6* (n = 1), *IGH*::*CDK6* (n = 1), *IGH*::*ASCL2* (n = 1), *IGH*::*PAX5* (n = 1) and *IGH*::*CCND3* (n = 1). Additionally, we screened 130 samples for *IGH*::*TERT*-translocation and identified one *IGH*::*TERT*-translocation and 90 samples for *IGH*::*BCL11A*-translocation and identified five *IGH*::*BCL11A*-translocations only by FISH. In summary, we detected the translocation partner in 50 *IG*-translocations (44 by NGS and 6 by FISH) with recurrent translocations in *BCL11A* (n = 12)*, METRNL* (n = 4)*, NKX2.6* (n = 3)*, MMSET* (n = 3)*, TERT* (n = 3)*, ZCCHC24* (n = 3)*, PVT1* (n = 2)*, CCND3* (n = 2) and *POU2AF1* (n = 2).

**Investigation of the class-switch recombination on the non-translocated allele**

Interestingly, 3/6 patient samples with translocation partners involving the cell-cycle show class-switch recombination (CSR) on the non-translocated allele (2x *CCND3*, 1x *CDK6*). For the transcription factor/polycomb repressive complex-related genes, 4/23 patient samples show class-switch on the non-translocated allele (3x *BCL11A*, 1x *BMI1*). All patient samples with translocations in the telomerase-associated genes show no class-switch in the non-translocated allele. In the *MYC-*associated genes, 2/4 patient samples show class-switch in the non-translocated allele (1x*PVT1*, 1x*MYCN*). 4/8 patient samples, belonging to the others group, show class-switch in the non-translocated allele (2x *METRNL*, 1x *ZCCHC24*, 1x*F13A1*).

**Expression of translocation partner genes in CLL with *IGH*-translocation**

For the *MYC*-associated group, we did not detect upregulation of *MYC* in one case with *IGH*::*PVT1*-translocation. For the *IGH*::*MMSET* translocation, it seems that both *MMSET* and *FGFR3* are upregulated (see Supplementary Figure 7A-E).

**Characterization of *IGH*::*BCL11A*-translocation in CLL**

From 7/12 cases, the exact breakpoint locations were identified using NGS, and the resulting fusions were verified in three cases by PCR-based Sanger sequencing. All identified breakpoints on chromosome 2 were located centromeric to the *BCL11A* gene (approximately 250bp distant from the putative transcriptional start site). On chromosome 14, breakpoints clustered within the switch regions of *IGHM* (n=4), *IGHG4* (n=2), *IGHG2* (n=2) and *IGHG1* (n=2) and between *IGHD2-2* and *IGHD1-1* (n=1) and between *IGHD1-1* and *IGHV6-1* (n=1) (see Figure 2A). N-nucleotides were not identified and one RSS site (RSS23) was identified in one case. The underlying translocation mechanism was determined to be aberrant CSR in five cases, aberrant VDJ recombination in one case and aberrant somatic hypermutation also in one case. Based on the location and underlying mechanisms, it is likely that at least one enhancer segment of the *IGH* locus remains on chromosome 14, consistent with the activation of the target oncogene *BCL11A* on chromosome 2. Analysis of *IGHV* mutation status, copy number variations (CNVs), and mutations in recurrently mutated genes among the twelve *IGH*::*BCL11A*-translocated CLL revealed the presence of unmutated *IGHV* in all cases. In addition, both CNVs and somatic mutations exhibited a highly heterogeneous distribution, with trisomy 12 detected in four cases (see Figure 2C).

**Analysis of co-occurrence of specific copy number aberrations and mutational landscape of CLL with *IGH*-translocation**

Expectedly, we identified significant co-occurrences between trisomy 12 and *NOTCH1* mutations (p < 0.05; OR: 3), as well as between deletion 17p and *TP53* mutations (p < 0.001; OR: 85).

**Comparison of** **CLL candidate gene mutation analysis from** **panel sequencing and WGS data**

The targeted sequencing data were compared with WGS data for 46 matching cases. This comparison revealed that 89% (48/54) of the variants identified in the targeted sequencing were also detected in the WGS data. However, there were some discrepancies between the two approaches. Variants detected only in targeted sequencing included *ATM* (NM_000051:exon2:c.67C>T), *EGR2* (NM_000399:exon2:c.1150C>A), *NOTCH1* (NM_017617:exon34:c.7541_7542del), and *SF3B1* (NM_012433: exon15:c.2117C>T). Additionally, two variants had low read coverage in WGS: *RPS15* (NM_001018:exon4:c.416C>G, detected in 1/27 reads) and *SF3B1* (NM_012433:exon14:c.1998G>C, detected in 3/38 reads). These low-coverage variants were only called as variants in the targeted sequencing approach.

**References:**

1. Danecek P, Bonfield JK, Liddle J, Marshall J, Ohan V, Pollard MO, et al. Twelve years of SAMtools and BCFtools. GigaScience. 2021 Feb 1;10(2):giab008.

2. Vasimuddin Md, Misra S, Li H, Aluru S. Efficient Architecture-Aware Acceleration of BWA-MEM for Multicore Systems. In: 2019 IEEE International Parallel and Distributed Processing Symposium (IPDPS). 2019. p. 314–24.

3. Andrews S. FastQC: a quality control tool for high throughput sequence data [Internet]. 2010 [cited 2024 Feb 21]. Available from: https://www.bioinformatics.babraham.ac.uk/projects/fastqc/

4. Nadeu F, Mas-de-les-Valls R, Navarro A, Royo R, Martín S, Villamor N, et al. IgCaller for reconstructing immunoglobulin gene rearrangements and oncogenic translocations from whole-genome sequencing in lymphoid neoplasms. Nature communications. 2020;11(1):3390.

5. Robinson JT, Thorvaldsdottir H, Turner D, Mesirov JP. igv.js: an embeddable JavaScript implementation of the Integrative Genomics Viewer (IGV). Bioinformatics. 2023 Jan 1;39(1):btac830.

6. Lee AI, Fugmann SD, Cowell LG, Ptaszek LM, Kelsoe G, Schatz DG. A functional analysis of the spacer of V(D)J recombination signal sequences. PLoS Biol. 2003 Oct;1(1):E1.

7. Cowell LG, Davila M, Kepler TB, Kelsoe G. Identification and utilization of arbitrary correlations in models of recombination signal sequences. Genome Biol. 2002;3(12):RESEARCH0072.

8. Cowell LG, Davila M, Ramsden D, Kelsoe G. Computational tools for understanding sequence variability in recombination signals. Immunol Rev. 2004 Aug;200:57–69.

9. Tausch E, Beck P, Schlenk RF, Jebaraj BJ, Dolnik A, Yosifov DY, et al. Prognostic and predictive role of gene mutations in chronic lymphocytic leukemia: results from the pivotal phase III study COMPLEMENT1. haematol. 2020 Oct 1;105(10):2440–7.

10. Tausch E, Schneider C, Robrecht S, Zhang C, Dolnik A, Bloehdorn J, et al. Prognostic and predictive impact of genetic markers in patients with CLL treated with obinutuzumab and venetoclax. Blood. 2020 Jun 25;135(26):2402–12.

11. Koboldt DC, Zhang Q, Larson DE, Shen D, McLellan MD, Lin L, et al. VarScan 2: somatic mutation and copy number alteration discovery in cancer by exome sequencing. Genome Res. 2012 Mar;22(3):568–76.

12. Knisbacher BA, Lin Z, Hahn CK, Nadeu F, Duran-Ferrer M, Stevenson KE, et al. Molecular map of chronic lymphocytic leukemia and its impact on outcome. Nature genetics. 2022;

13. Kröber A, Seiler T, Benner A, Bullinger L, Brückle E, Lichter P, et al. V(H) mutation status, CD38 expression level, genomic aberrations, and survival in chronic lymphocytic leukemia. Blood. 2002 Aug 15;100(4):1410–6.

14. Jaramillo S, Agathangelidis A, Schneider C, Bahlo J, Robrecht S, Tausch E, et al. Prognostic impact of prevalent chronic lymphocytic leukemia stereotyped subsets: analysis within prospective clinical trials of the German CLL Study Group (GCLLSG). haematol. 2020 Nov 1;105(11):2598–607.

15. Dobin A, Davis CA, Schlesinger F, Drenkow J, Zaleski C, Jha S, et al. STAR: ultrafast universal RNA-seq aligner. Bioinformatics. 2013 Jan 1;29(1):15–21.

16. Pertea M, Pertea GM, Antonescu CM, Chang TC, Mendell JT, Salzberg SL. StringTie enables improved reconstruction of a transcriptome from RNA-seq reads. Nat Biotechnol. 2015 Mar;33(3):290–5.

17. Koldobskiy MA, Jenkinson G, Abante J, Rodriguez DiBlasi VA, Zhou W, Pujadas E, et al. Converging genetic and epigenetic drivers of paediatric acute lymphoblastic leukaemia identified by an information-theoretic analysis. Nat Biomed Eng. 2021 Apr;5(4):360–76.

18. Kushwaha G, Dozmorov M, Wren JD, Qiu J, Shi H, Xu D. Hypomethylation coordinates antagonistically with hypermethylation in cancer development: a case study of leukemia. Hum Genomics. 2016 Jul 25;10 Suppl 2(Suppl 2):18.

19. Love MI, Huber W, Anders S. Moderated estimation of fold change and dispersion for RNA-seq data with DESeq2. Genome Biology. 2014 Dec 5;15(12):550.

20. Uhrig S, Ellermann J, Walther T, Burkhardt P, Fröhlich M, Hutter B, et al. Accurate and efficient detection of gene fusions from RNA sequencing data. Genome Res. 2021 Mar;31(3):448–60.

21. R Core Team (2024) _R. A Language and Environment for Statistical Computing_. R Foundation for Statistical Computing. In Vienna, Austria; Available from: https://www.R-project.org/>.

22. Hao Y, Stuart T, Kowalski MH, Choudhary S, Hoffman P, Hartman A, et al. Dictionary learning for integrative, multimodal and scalable single-cell analysis. Nature Biotechnology. 2024 Feb 1;42(2):293–304.

23. Massoni-Badosa R, Aguilar-Fernández S, Nieto JC, Soler-Vila P, Elosua-Bayes M, Marchese D, et al. An atlas of cells in the human tonsil. Immunity. 2024 Feb 13;57(2):379-399.e18.

24. Satterwhite E, Sonoki T, Willis TG, Harder L, Nowak R, Arriola EL, et al. The BCL11 gene family: involvement of BCL11A in lymphoid malignancies. Blood. 2001 Dec 1;98(12):3413–20.

25. Liu H, Ippolito GC, Wall JK, Niu T, Probst L, Lee BS, et al. Functional studies of BCL11A: characterization of the conserved BCL11A-XL splice variant and its interaction with BCL6 in nuclear paraspeckles of germinal center B cells. Mol Cancer. 2006 May 16;5:18.
