# Supplementary material for: The spectrum of immunoglobulin heavy chain enhancer hijacking in chronic lymphocytic leukemia
Source: Leukemia. 2026 Apr 23;40(7):1401–10. doi: 10.1038/s41375-026-02902-9 (PMC13323050; doi:10.1038/s41375-026-02902-9)
Supplement: Supplementary file 3 — Supplementary Figures [file 41375_2026_2902_MOESM3_ESM.pdf]

Supplementary Figure 1:

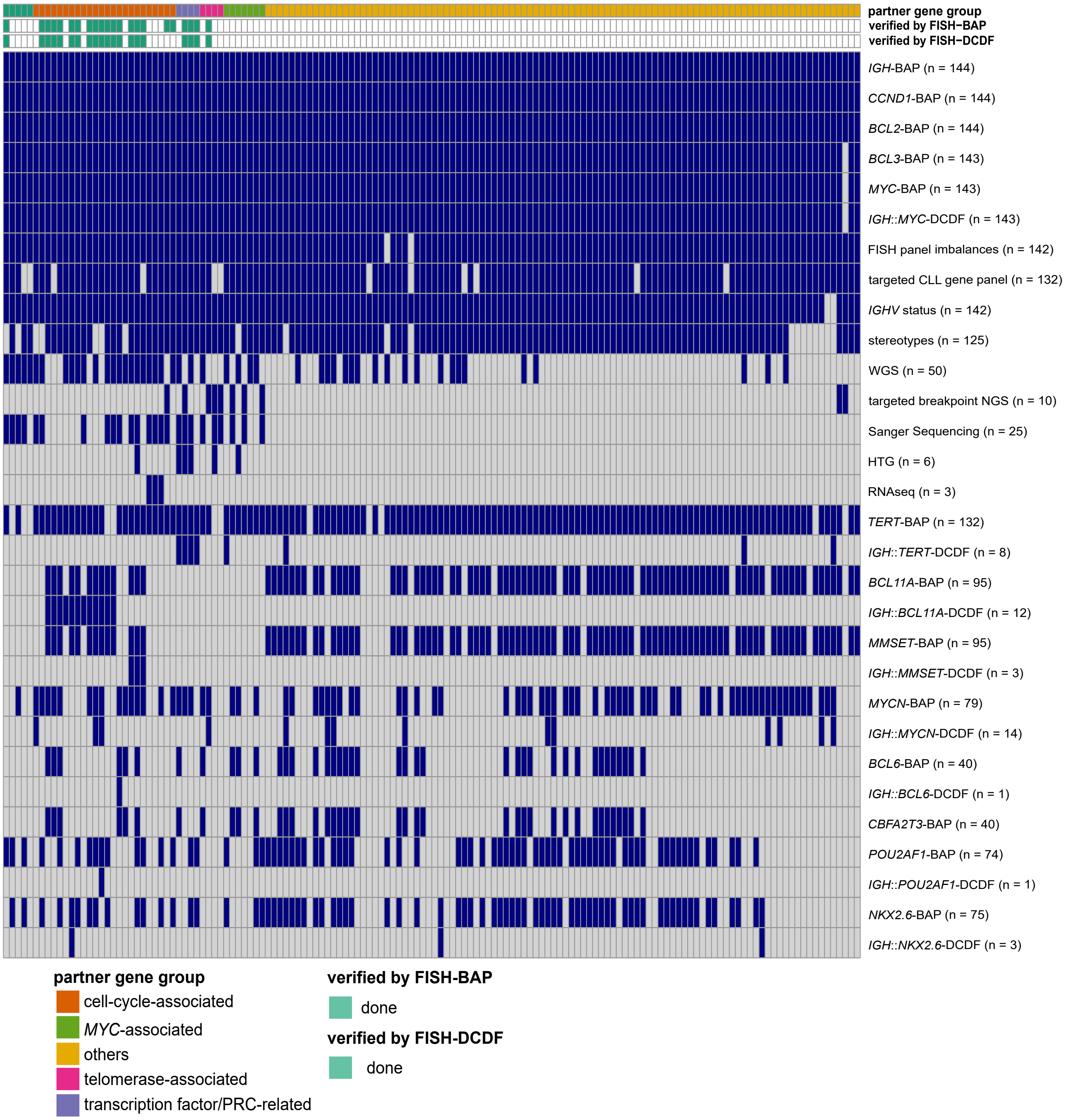

Supplementary Figure 1: Number of cases and the used methods. Heatmap of all the used methods and their corresponding number of cases (on the right). Samples are sorted according to the translocation group and partner gene (annotation on the top). The 25 genes can be separated in five different groups: cell-cycle genes, transcription factors/polycomb repressive complex (PRC) genes, telomerase-associated genes, *MYC*-associated genes, others. BAP: break-apart probe; DCDF: dual colour dual fusion; FISH: fluorescence in-situ hybridisation; NGS: next generation sequencing; WGS: whole genome sequencing; CLL: chronic lymphocytic leukemia, PRC: polycomb repressive complex.

Supplementary Figure 2:

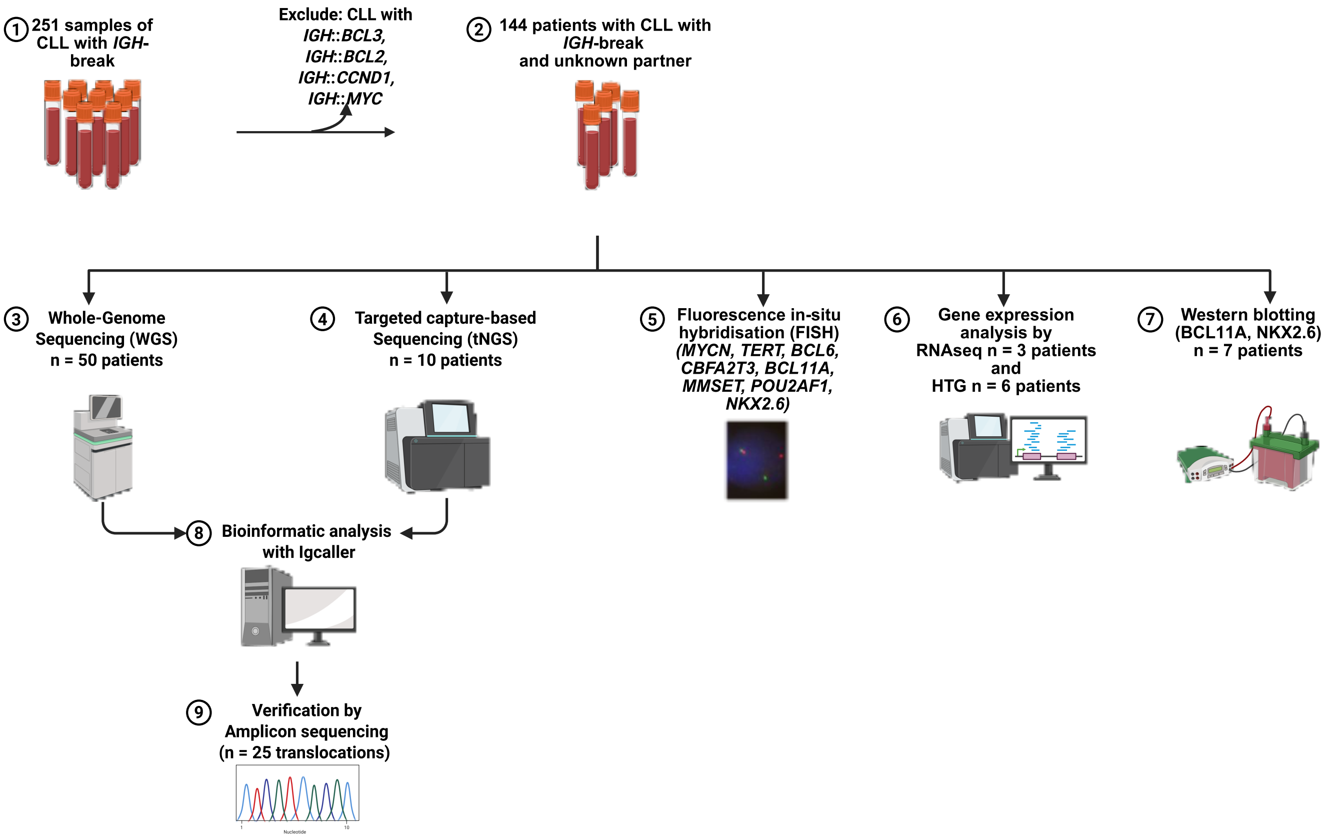

Supplementary Figure 2: Workflow of the selection of CLL with *IGH*-translocation for the identification of new translocation partner genes. CLL: chronic lymphocytic leukemia; *IGH*: immunoglobulin heavy chain.

Supplementary Figure 3:

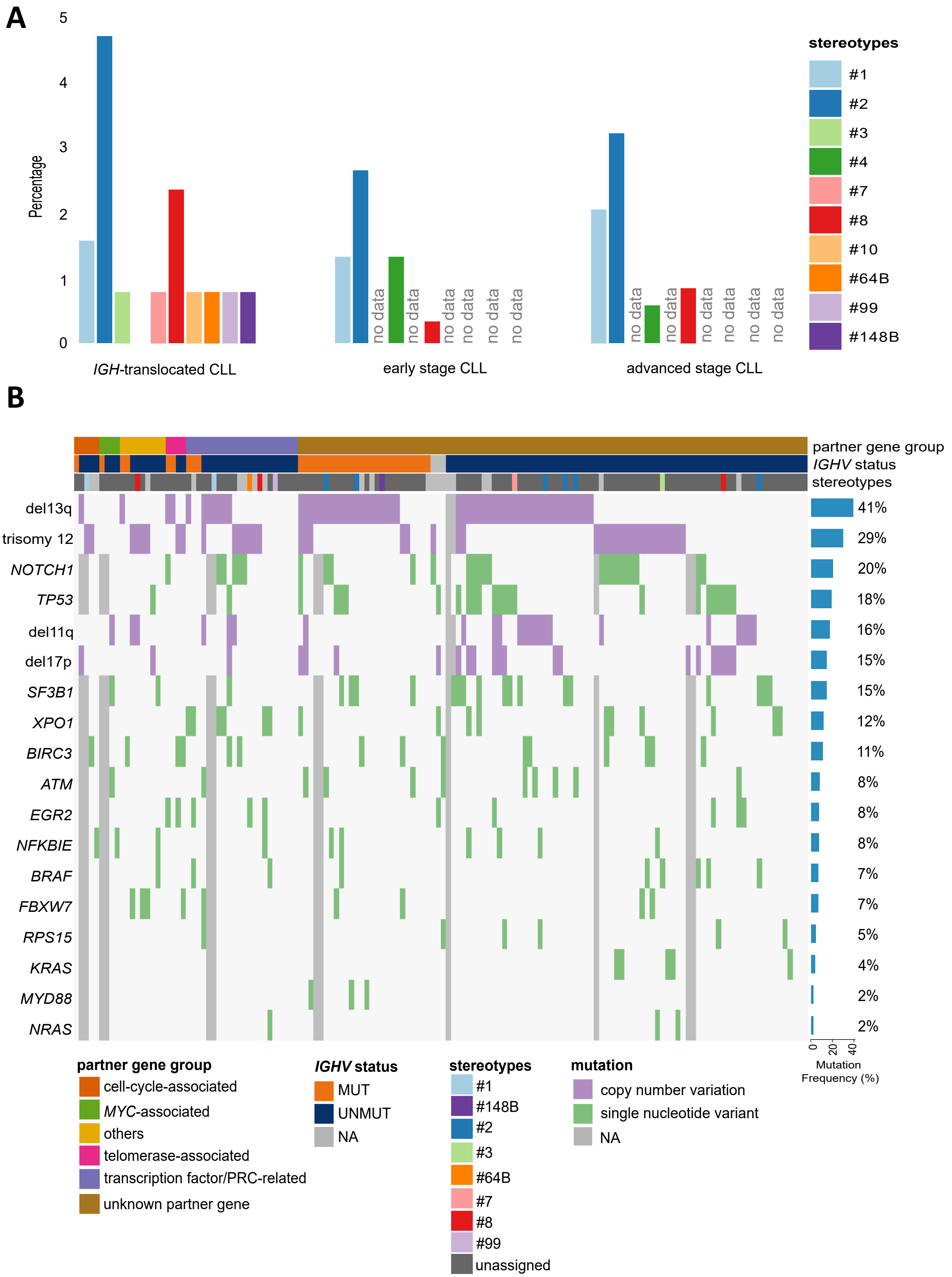

Supplementary Figure 3: Genomic characterization of the *IGH*-translocated CLL. A: Barplot of the percentage of different stereotypes in *IGH*-translocated CLL (n = 125; left panel) compared to early (n = 592; middle panel, Jaramillo et al., 2020) and advanced stage CLL (n = 1861; right panel, Jaramillo et al., 2020). **B:** Oncoplot of recurrently mutated genes (in green) and copy number variations (in purple) in CLL with *IGH*-translocation (n = 144). Top annotations show classification, partner gene group, *IGHV* mutation status and stereotypes and are sorted according to the partner gene groups. The aberrations on the y-axis are sorted according to their frequency. NA: not available, PRC: polycomb repressive complex.

Supplementary Figure 4:

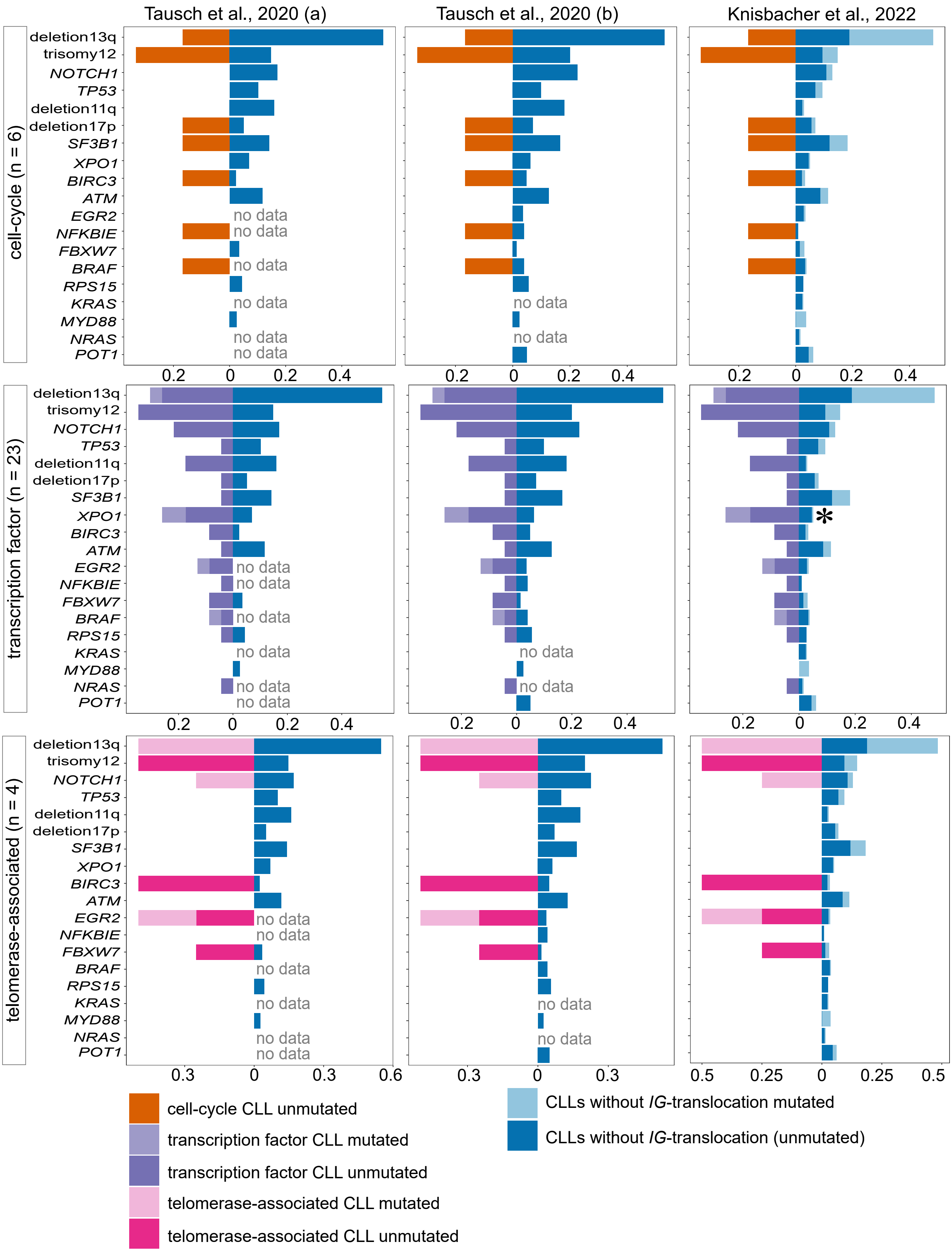

Supplementary Figure 4: Analysis of copy number aberration and mutational landscape of CLL with *IGH*-translocation for the cell-cycle, transcription factor and telomerase-asscoaited group. Barplots of percentage of different recurrently mutated genes and copy number variations in *IGH*-translocated CLL from the defined translocation partner groups (cell-cycle, transcription factor and telomerase-associated) and data from three different CLL populations as control. * = FDR < 0.05.

Supplementary Figure 5:

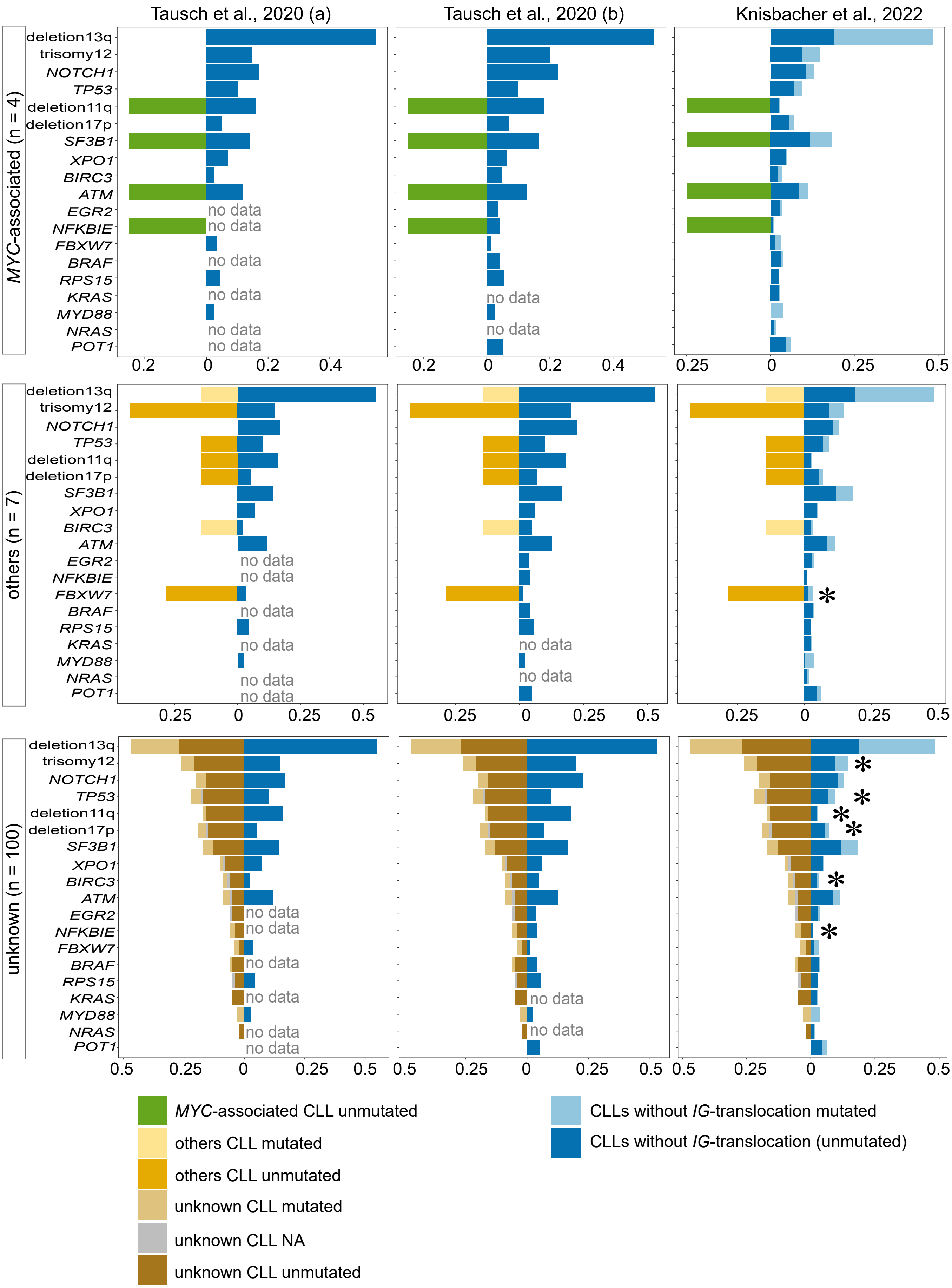

Supplementary Figure 5: Analysis of copy number aberration and mutational landscape of CLL with *IGH*-translocation for the *MYC*-associated, others and unknown group. Barplots of percentage of different recurrently mutated genes and copy number variations in *IGH*-translocated CLL from the defined transcloation partner groups (*MYC*-associated, others, unknown) and data from three different CLL populations as control. * = FDR < 0.05, MUT = mutated *IGHV*, UNMUT = unmutated *IGHV*.

Supplementary Figure 6:

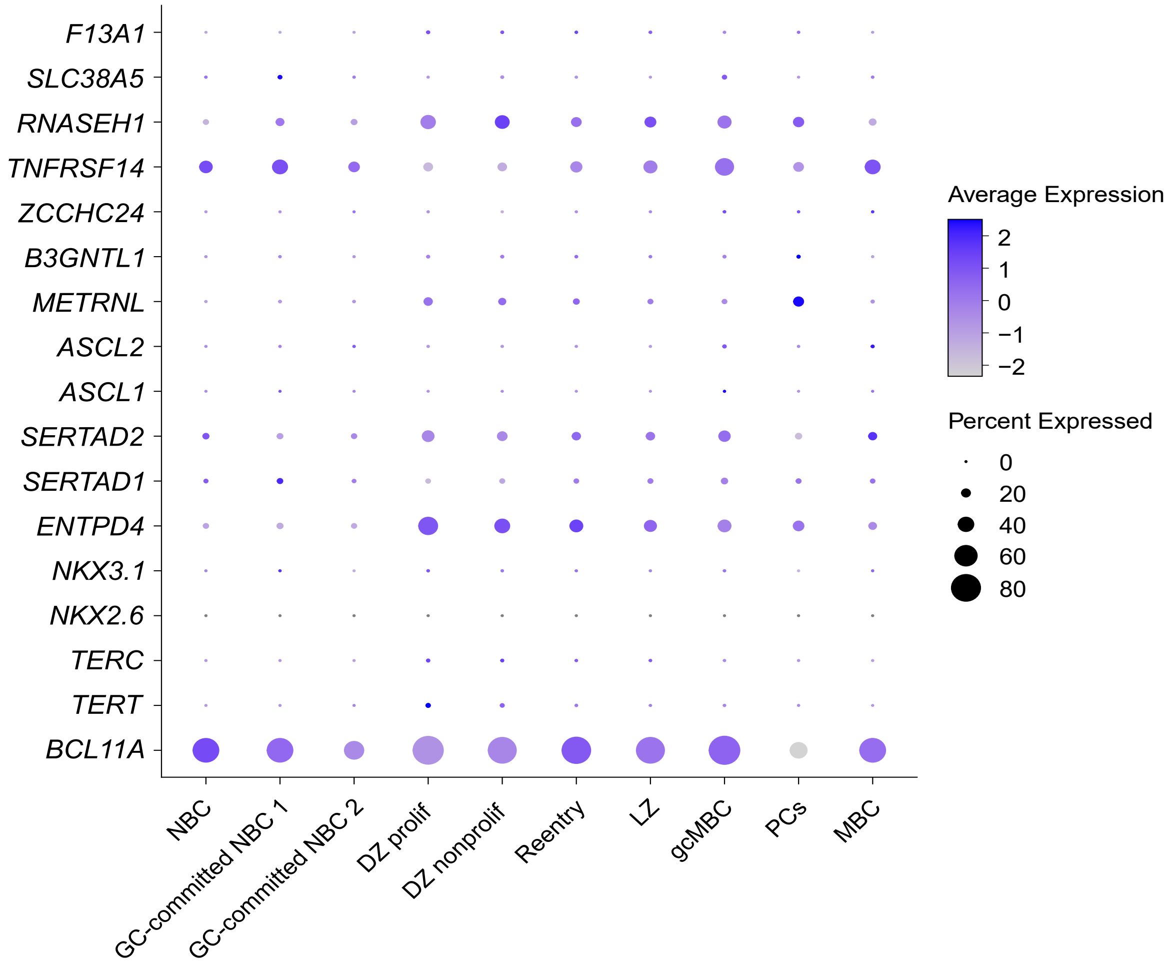

Supplementary Figure 6: Single-cell RNA-sequencing analysis of human tonsil reference. Single cell RNA expression data (Massoni-Badosa et al., 2024) of selected genes (y-axis) during B-cell development (x-axis). Purple colour represents the average expression levels and size of the dots represents the precentage of expressed cells. NBC: naive B-cell, DZ prolif: dark zone proliferating, DZ nonprolif: dark zone nonproliferating, LZ: light zone, GC: germinal center, gcMBC: germinal center memory B-cells, MBC: memory B-cells, PCs: plasma cells.

Supplementary Figure 7:

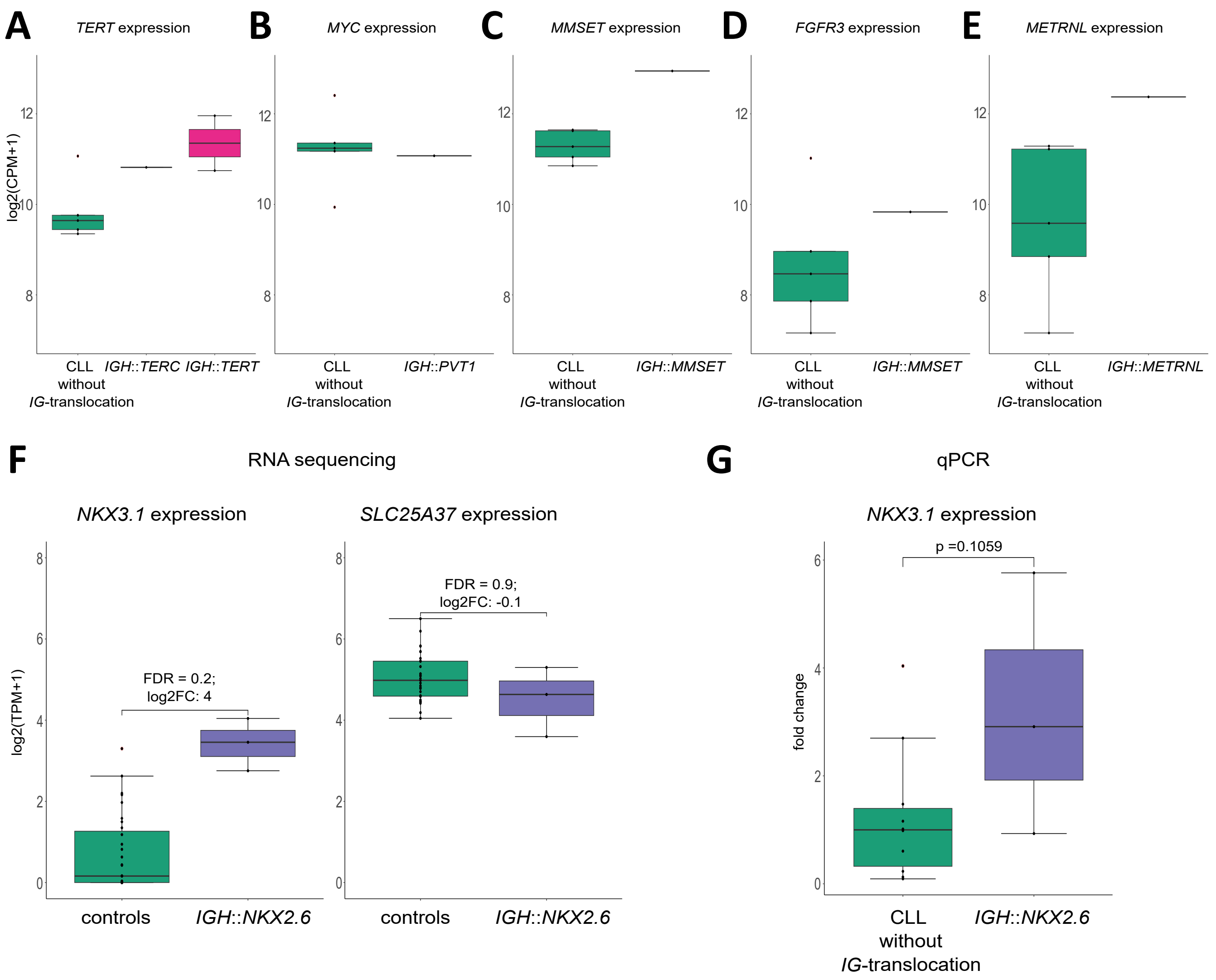

Supplementary Figure 7: Gene expression analysis of selected *IGH*-translocated CLL. Boxplots with different *IGH*-translocated CLL and CLL without *IG*-translocation as controls on x-axis and log2(CPM+1) on the y-axis showing the *TERT* **(A)**, *MYC* **(B)**, *MMSET* **(C)**, *FGFR3* **(D)** and *METRNL* **(E)** expression. Due to small sample size, significance was not calculated. **F:** *NKX3.1* and *SLC25A37* expression in RNA sequencing data of *IGH*::*NKX2.6*-translocated CLL (n = 3) compared to controls (n = 30). **G:** Boxplots from qPCR data of *IGH*::*NKX2.6*-translocated CLL (n = 3) and CLLs without *IG*-translocation (n = 10) on x-axis and fold change on the y-axis showing the upregulation of *NKX3.1*.

Supplementary Figure 8:

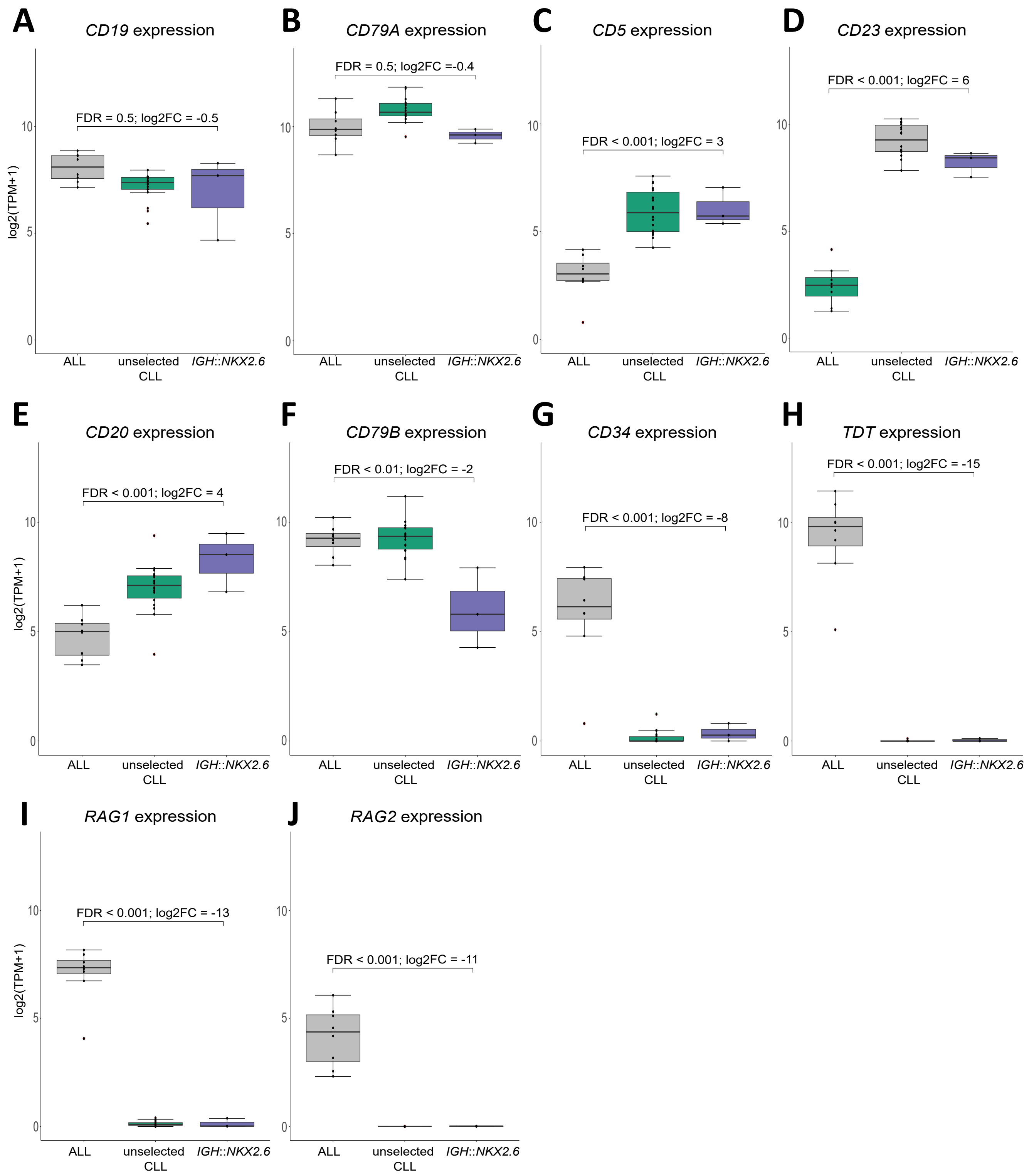

Supplementary Figure 8: Comparison of gene expression in ALL, CLL and *IGH*::*NKX2.6*-translocated CLL. Boxplots with ALL (Koldobskiy et al., 2021), unselected CLL (Kushwaha et al., 2016) and *IGH*::*NKX2.6*-translocated CLL on x-axis and log2(TPM+1) on the y-axis showing the *CD19* **(A)**, *CD79A* **(B)**, *CD5* **(C)**, *CD23* **(D)**, *CD20* **(E)**, *CD79B* **(F)**, *CD34* **(G)**, *TDT* **(H)**, *RAG1* **(I)** and *RAG2* **(J)** expression.

Supplementary Figure 9:

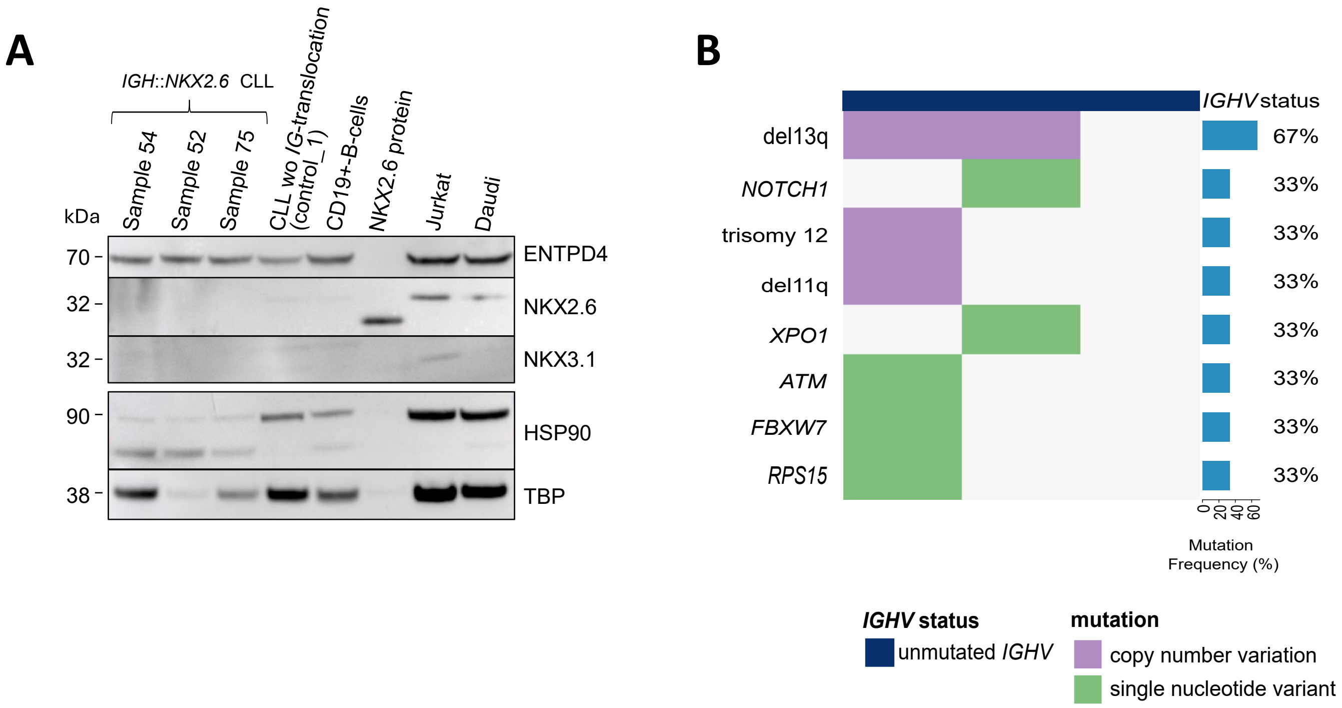

Supplementary Figure 9: Characterization of *IGH*::*NKX2.6*-translocated CLL by protein expression and mutation analysis. A: Western Blot of the protein expression of NKX2.6, NKX3.1 and ENTPD4 and the housekeeper genes HSP90 and TBP for *IGH*::*NKX2.6*-translocated CLL (n = 3), one CLL without *IG*-translocation, one sample of CD19+ B-cells, one samples of NKX2.6 protein and Jurkat and Daudi cell lines. **B:** Oncoplot of recurrently mutated genes (in green) and copy number variations (in purple) in CLL with *IGH*::*NKX2.6*-translocation. Top annotation show *IGHV* mutation status. The aberrations on the y-axis are sorted according to their frequency.

Supplementary Figure 10:

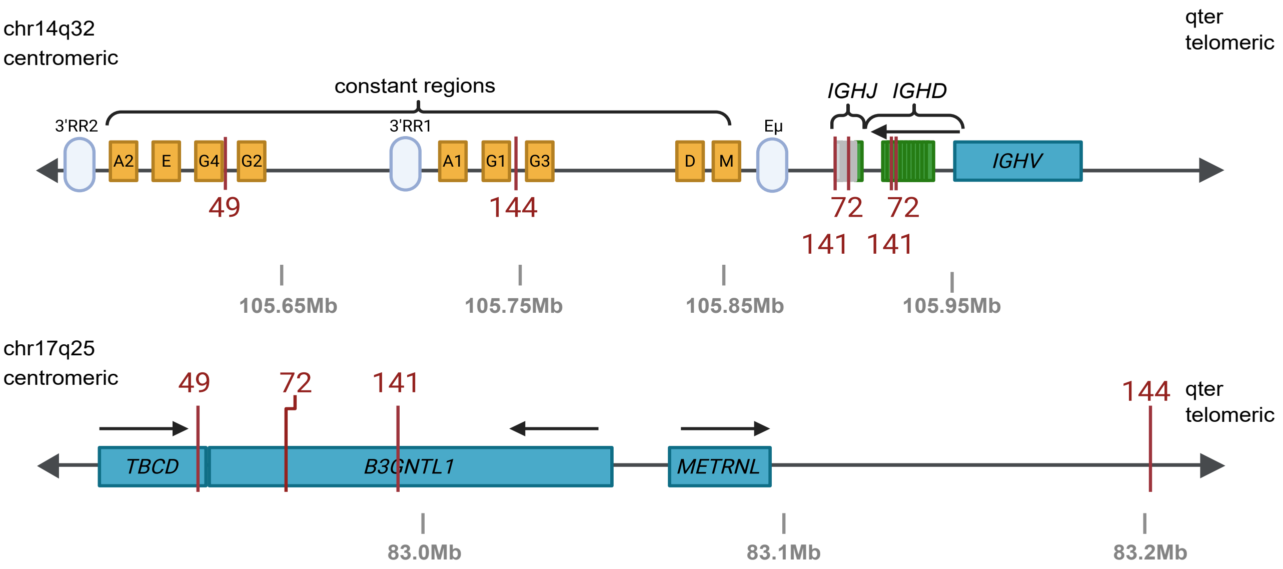

Supplementary Figure 10: Characterization of *IGH*::*METRNL*-translocated CLL. *IGH*-breakpoints and breakpoints in chromosome 17q25 of the four samples with CLL (one line belongs to two breakpoints in chromosome 17) with *IGH*::*METRNL*-translocation.

Supplementary Figure 11:

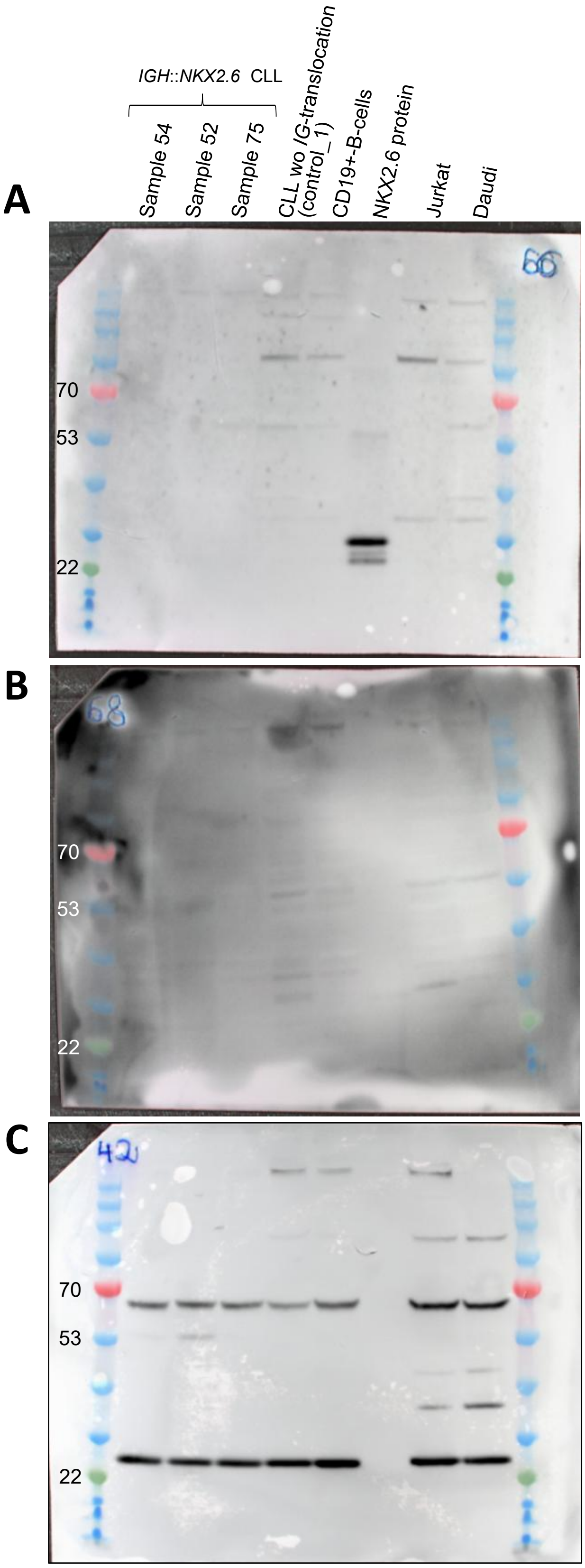

Supplementary Figure 11: Western Blots. Western Blots of NKX2.6 (A), NKX3.1 (B) and ENTPD4 (C).
